# The TBC/RabGAP Armus Coordinates Rac1 and Rab7 Functions during Autophagy

**DOI:** 10.1016/j.devcel.2013.03.005

**Published:** 2013-04-15

**Authors:** Bernadette Carroll, Noor Mohd-Naim, Filipe Maximiano, Marieke A. Frasa, Jessica McCormack, Mattea Finelli, Sigrid B. Thoresen, Louis Perdios, Reiko Daigaku, Richard E. Francis, Clare Futter, Ivan Dikic, Vania M.M. Braga

**Affiliations:** 1 Molecular Medicine, NHLI, Faculty of Medicine, Imperial College London, London SW7 2AZ, UK; 2 Institute of Ophthalmology, University College London, London EC1V 9EL, UK; 3 Institute of Biochemistry II, Goethe University, Frankfurt D-60590, Germany

## Abstract

Autophagy is an evolutionarily conserved process that enables catabolic and degradative pathways. These pathways commonly depend on vesicular transport controlled by Rabs, small GTPases inactivated by TBC/RabGAPs. The Rac1 effector TBC/RabGAP Armus (TBC1D2A) is known to inhibit Rab7, a key regulator of lysosomal function. However, the precise coordination of signaling and intracellular trafficking that regulates autophagy is poorly understood. We find that overexpression of Armus induces the accumulation of enlarged autophagosomes, while Armus depletion significantly delays autophagic flux. Upon starvation-induced autophagy, Rab7 is transiently activated. This spatiotemporal regulation of Rab7 guanosine triphosphate/guanosine diphosphate cycling occurs by Armus recruitment to autophagosomes via interaction with LC3, a core autophagy regulator. Interestingly, autophagy potently inactivates Rac1. Active Rac1 competes with LC3 for interaction with Armus and thus prevents its appropriate recruitment to autophagosomes. The precise coordination between Rac1 and Rab7 activities during starvation suggests that Armus integrates autophagy with signaling and endocytic trafficking.

## Graphical abstract

**Graphical Abstract:**

**Figure:**
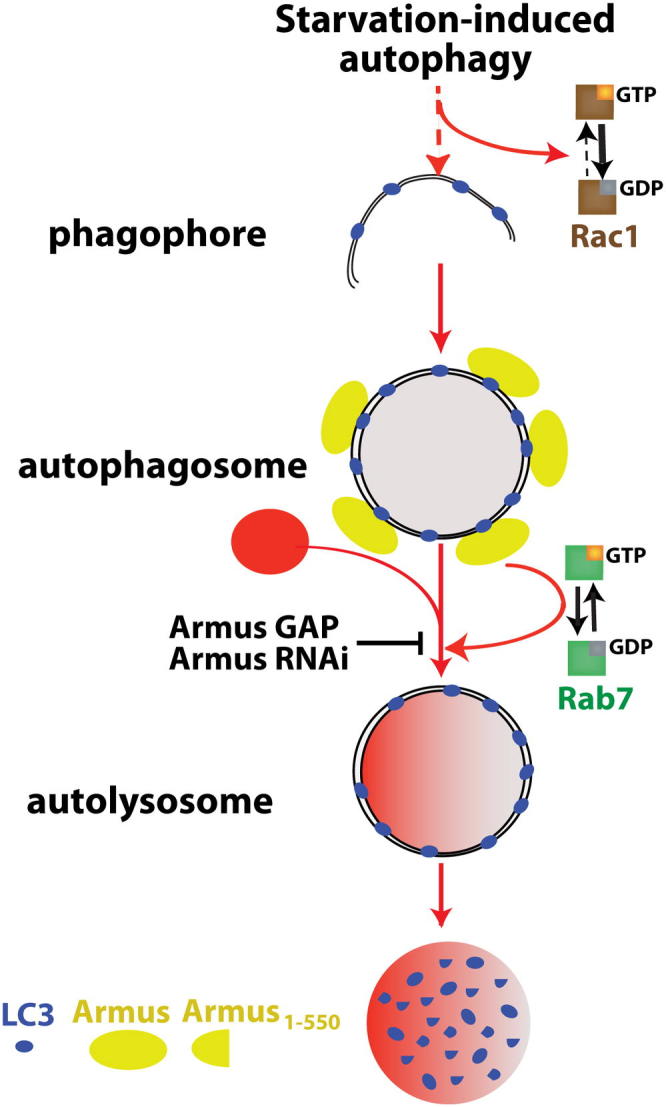

**Highlights:**

► Autophagy strongly inactivates Rac1 GTPase and recruits the TBC/RabGAP Armus ► Appropriate Armus localization at autophagosomes requires binding to LC3 ► Armus inhibition delays autophagic flux and increases levels of Rab7·GTP ► Rac1, Armus, and Rab7 coordinate efficient lysosome fusion with autophagosomes

## Teaser

The Rac1 effector Armus is a GTPase-activating protein that regulates Rab7 function and thus endosomal trafficking to lysosomes. Carroll et al. find that Armus binds directly to the core autophagy factor LC3, facilitates fusion of autophagosomes with lysosomes, and integrates local Rac1 activity into the control of autophagy.

## Introduction

Autophagy is a fundamental process involved in homeostasis, cell survival, and differentiation, among other processes. Autophagy can be triggered by different stimuli such as differentiation (i.e., mitophagy), deprivation of amino acids (starvation induced), or during homeostasis (basal autophagy) (Levine and Kroemer, 2008). Different types of autophagy share a core machinery and result in degradation of unwanted intracellular material, yet they have common (Webber and Tooze, 2010) and distinct regulators (Chan et al., 2007; Lee et al., 2010; Nishida et al., 2009; Underwood et al., 2010). While autophagy is tightly regulated in its own right (Chen and Klionsky, 2011; Klionsky, 2007; Ravikumar et al., 2010b), it requires integration with intracellular trafficking and signaling pathways regulating the cytoskeleton, differentiation, or anabolic/catabolic processes. However, the molecular mechanisms that coordinate these diverse signaling pathways during autophagy are unknown (Chen and Klionsky, 2011).

A complex network of core components (autophagy-related or Atg proteins) controls the initiation and maturation of autophagosomes by recruiting proteins required for membrane elongation, movement, and fusion with a number of vesicular compartments. Among the core proteins, Atg8/LC3 (microtubule-associated light chain 3) is essential for expansion/fusion of membranes to form autophagosomes (Longatti and Tooze, 2009; Nakatogawa et al., 2007; Tooze, 2010). Ultimately, autophagosome contents are degraded upon fusion with lysosomes (i.e., autolysosomes) (Levine and Kroemer, 2008; Longatti and Tooze, 2009; Tooze, 2010).

Rab GTPases regulate intracellular trafficking, such as budding, transport, and fusion of vesicles with distinct vesicular compartments, cell membranes, or intracellular organelles. A number of Rabs have been shown to regulate autophagosome biogenesis: Rab1 (Huang et al., 2011; Zoppino et al., 2010), Rab11 (Fader et al., 2008; Longatti et al., 2012), Rab7 (Gutierrez et al., 2004; Jäger et al., 2004), Rab9 (Nishida et al., 2009), and Rab33 (Itoh et al., 2008). Importantly, Rabs may regulate the intracellular movement of autophagosomes required for their maturation (Jäger et al., 2004; Korolchuk et al., 2011; Ravikumar et al., 2010a). The ability of LC3 to recruit Rab regulators, effectors, and partners to autophagosomes indicates that LC3 may act as an organizer and scaffolding protein (Behrends et al., 2010; Itoh et al., 2011; Pankiv et al., 2010; Popovic et al., 2012).

How Rab function is coordinated during fusion of different endomembranes with autophagosomes remains unclear (Stenmark, 2009). A large number of Rabs may be involved in autophagy, and each cycle of Rab activation/inactivation is precisely controlled. Both positive (exchange factors, or GEFs) and negative (GTPase-activating proteins, or GAPs) regulators of Rabs define the timing, duration, and specificity of Rab signaling at a particular intracellular compartment (Stenmark, 2009). Rab GAPs contain the highly conserved TBC domain (Tre2/Bub2/Cdc16) that inactivates Rabs by facilitating the hydrolysis of Rab-associated guanosine triphosphate (GTP) into guanosine diphosphate (GDP) (Frasa et al., 2012). Different TBC-containing RabGAPs have been shown to interact with LC3 and may integrate autophagy with intracellular trafficking (Behrends et al., 2010; Itoh et al., 2011; Longatti et al., 2012; Popovic et al., 2012). However, the specific steps regulated by most TBC/RabGAPs during autophagosome biogenesis are not known.

The TBC/RabGAP Armus (TBC1D2A, isoform 1; Uniprot accession number Q9BYX2-1) specifically inactivates Rab7, a Rab required for lysosome function (Frasa et al., 2010). Armus is also an effector of Rac1 (Frasa et al., 2010), a small GTPase that regulates cytoskeletal remodeling, migration, and adhesion events (Mack et al., 2011). Upon epidermal growth factor (EGF) treatment, Armus regulates E-cadherin degradation during cell scattering but has no effect on cadherin levels at steady state. Armus mediates a crosstalk between Rac1 activation and Rab7 cycling (Frasa et al., 2010) and thus coordinates the function of these two small GTPases during cell scattering.

Here, we set out to test the hypothesis that Armus provides a signaling node for the localized activation/inactivation of Rab7 during autophagy. Rab7 is clearly required for autolysosome formation (Gutierrez et al., 2004; Jäger et al., 2004); however, it remains unclear whether or how Rac1 might affect autophagy. We demonstrate that Armus and its partners Rac1 and Rab7 participate in both basal and starvation-induced autophagy in unexpected ways. In contrast to E-cadherin degradation, we show that upstream regulation of Armus in autophagy does not require Rac1 activation. Rather, upon starvation, Rac1 is strongly inactivated, while Rab7 is transiently activated. Our findings define molecular mechanisms to integrate signaling from distinct classes of GTPases to regulate autophagosome biogenesis.

## Results

### Armus Expression Induces Autophagosome Accumulation

**Figure 1:**
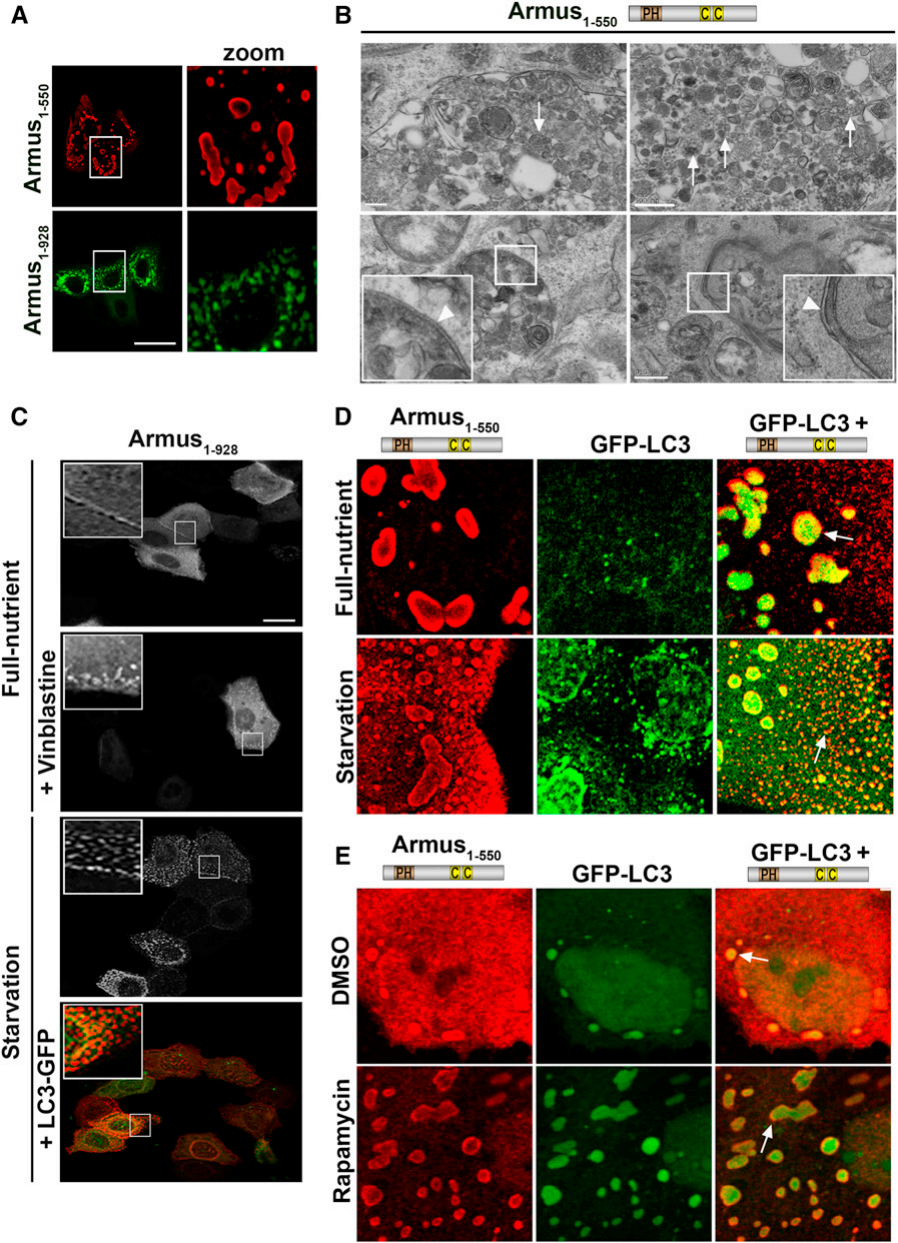
Armus Expression Induces Accumulation of Autophagosomes (A) Full-length Armus (Venus-Armus_1–928_) or its N-terminal region (myc-Armus_1–550_) was microinjected and expressed for 3 or 5 hr in full-nutrient medium. (B) Armus_1–550_ was injected by itself and cells processed for electron microscopy. Arrows show additional vesicles inside Armus autophagosomes; arrowheads point to double membranes of phagophores. Scale bar represents 200 μm (left panels) or 500 μm (right panels). (C) Full-length Armus (RFP-Armus_1–928_) was expressed at low levels by itself or in combination with GFP-LC3. Cells were treated with vehicle or vinblastine (50 μM) or starved in amino-acid-deficient medium to induce autophagosomes. Scale bar represents 50 μm and 12 μm for zoom. (D and E) Armus_1–550_ and GFP-LC3 were expressed alone or in combination. Cells were kept in full-nutrient medium or autophagy was induced by (D) starvation for 30 min or (E) treatment with 20 μM rapamycin for 1 hr. Arrows show colocalization with LC3. Scale bar represents 4 μm or 16 μm for zooms (A and C) or 25 μm (D and E). Representative images and quantifications are from three independent experiments (thereafter n = 3). See also Figure S1.

Armus expression (N terminus [Armus_1–550_] or full-length [Armus_1–928_]) led to formation of numerous enlarged vesicles in full-nutrient medium (Figure 1A) (Frasa et al., 2010). Although Armus colocalized with E-cadherin (Frasa et al., 2010), enlarged vesicles do not contain E-cadherin complexes (Figure S1A available online). By electron microscopy, Armus-expressing cells showed enlarged vesicles containing a number of structures of different shapes and sizes as well as the presence of double membranes characteristic of phagophores, the autophagosome precursors (Figure 1B).

Three approaches were taken to confirm that these vesicles are indeed autophagosomes. First, full-length was expressed at low levels, so that no vesicles were observed in controls (Figure 1C). Vinblastine treatment (which disrupts microtubules and vesicle movement) triggered Armus_1–928_ accumulation into small puncta. Second, amino acid starvation of cells expressing Armus (Figures 1C and 1D) or treatment with rapamycin (Figure 1E) showed de novo appearance of numerous autophagosomes that colocalized with GFP-LC3. Third, as an additional control, enlarged autophagosomes did not derive from Armus aggregation (Figure S1B). Armus labeled mostly the outer membrane of autophagosomes, while α-synuclein^A53T^, a mutant protein known to aggregate (Webb et al., 2003), formed small puncta in keratinocytes that were clearly distinct from Armus-induced vesicles (Figure S1B). Collectively, our data indicate that Armus may participate in basal and starvation-induced autophagy.

### Mechanisms of Autophagosome Accumulation by Armus in Full-Nutrient Medium

**Figure 2:**
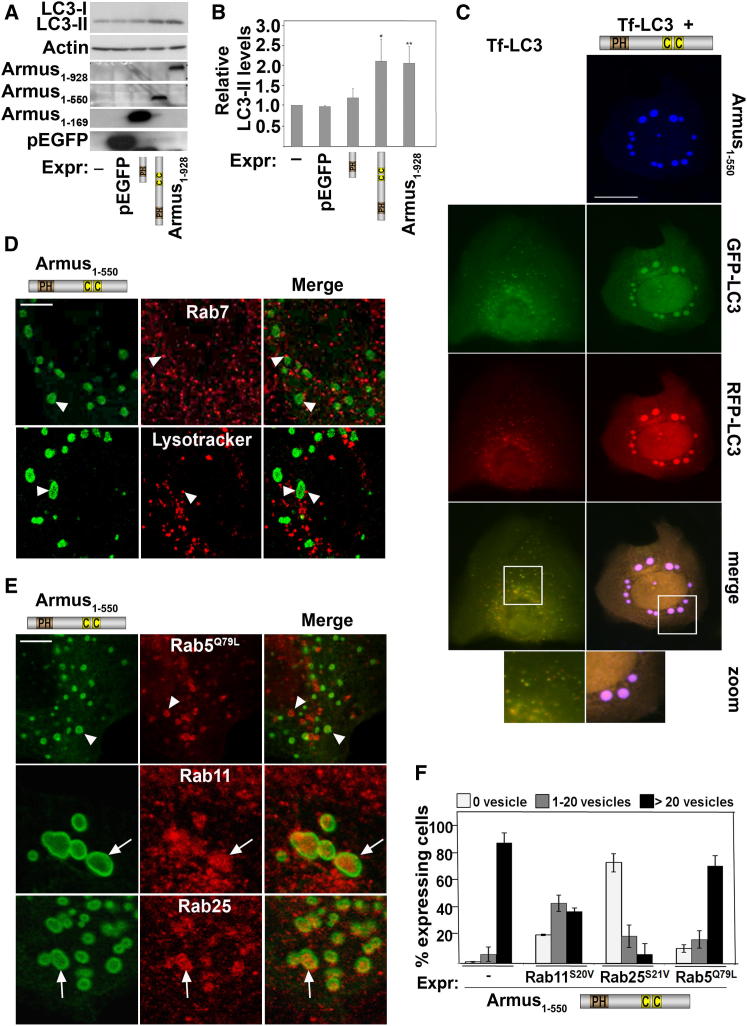
Autophagosomes Induced by Armus_1–550_ Require Late Endocytic Recycling, but Not Lysosomal Fusion (A) Keratinocytes were transfected with empty vector, flag-Armus_1–169_, myc-Armus_1–550_, or Venus-Armus_1–928_ and lysates were probed with anti-LC3 and anti-epitope tags. (B) Levels of LC3 were quantified and expressed relative to mock-transfected cells. (C) Tandem-fluorescent LC3 (Tf-LC3, mRFP-EGFP-LC3) was expressed alone or in combination with Armus_1–550_. Merged images and zoom are shown at the bottom. (D) Myc-Armus_1–550_ was transfected and cells stained for endogenous Rab7 and the tag. Alternatively, cells were incubated with lysotracker for 30 min followed by 2 hr chase. (E) Myc-Armus_1–550_ was microinjected in combination with activated Rab5 (Rab5^Q79L^) and cells stained for the respective tags. In addition, Armus_1–550_ was injected by itself and cells stained for the myc-tag and endogenous Rab11 or Rab25. (F) Keratinocytes were microinjected with myc-Armus_1-550_ alone or in combination with GFP-tagged versions of constitutively active Rab5 (Rab5^Q79L^), Rab11 (Rab11^S20V^) or Rab25 (Rab25^S21V^). The percentage of expressing cells showing no vesicles, 1 to 20 vesicles or more than 20 vesicles was quantified for each condition. Arrows show colocalization with Armus; arrowheads point to distinct localization from Armus. Scale bars represent 4 μm (D and E) or 25 μm (C). n = 3; ^∗^p < 0.05; ^∗∗^p < 0.009. Error bars represent the SD. See also Figure S2.

Expression of Armus_1–928_ or Armus_1–550_ in full-nutrient medium significantly increased LC3 protein levels (Figures 2A and 2B), confirming that LC3 accumulated in autophagosomes (Figures 1D and 1E). Expression of tandem-fluorescent LC3 (Tf-LC3) with Armus N terminus resulted in autophagosomes labeled with all fluorophores (Figure 2C), indicating that fusion with acidic compartments did not occur to quench the GFP fluorescence of Tf-LC3 (Kimura et al., 2007). Furthermore, endogenous Rab7, lysotracker, and two other lysosomal proteins did not label enlarged Armus_1–550_ vesicles (Figure 2D; Figure S2A). Endogenous Rab11 and Rab25, markers of recycling endosomes, were found inside Armus-labeled vesicles (Figure 2E), but early endosomal markers were not (i.e., active Rab5 or transferrin; Figure 2E; Figure S2A). Rab11/Rab25 localization is functionally relevant, as inhibiting their cycling interfered with Armus-dependent vesicles (Figure 2E; Figure S2B). Taken together, these results strongly suggest that Armus_1–550_ may block fusion of autophagosomes with lysosomes in basal conditions and that enlarged vesicles contain recycling membranes.

**Figure 3:**
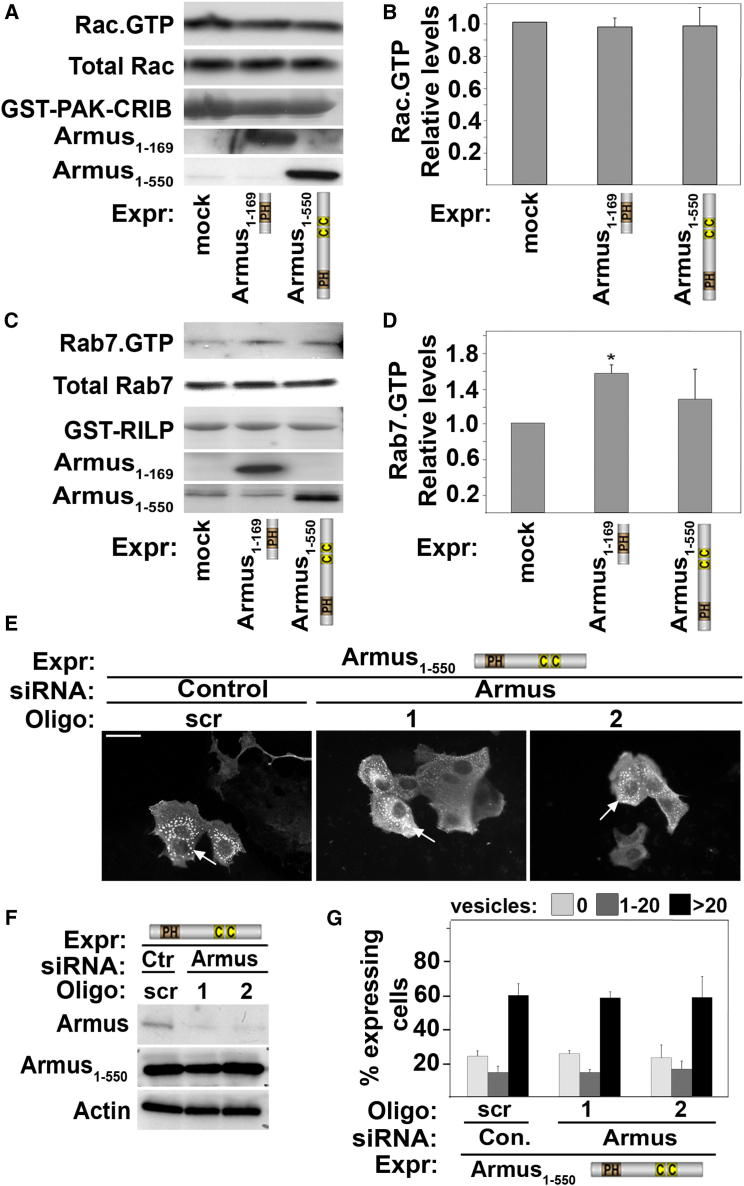
Mechanisms of Vesicle Accumulation by Armus_1–550_ Expression (A–D) Keratinocytes were transfected with empty vector, flag-Armus_1-169_ or myc-Armus_1-550_ (bottom of panels). Lysates were prepared and processed to detect active Rac1 (A and B) or active Rab7 (C and D). Values were expressed relative to mock-transfected cells (arbitrarily set as 1). Expression of the different constructs is detected by the respective tags and fusion protein levels are shown as amido black staining. (A) Active Rac1 (Rac·GTP) was determined using PAK-CRIB pull-down and probing with anti-Rac1. (B) Levels of endogenous Rac1 (Total Rac) were quantified to calculate the relative amount of active Rac1. (C) Lysates of cells transfected with wild-type GFP-Rab7 were incubated with GST-RILP to pull down active Rab7 (Rab7·GTP). (D) Levels of active Rab7 were calculated relative to total levels of GFP-Rab7. (E–G) Endogenous Armus was depleted in keratinocytes using two independent siRNA oligos (1 and 2). Cells were microinjected with Armus_1–550_ and fixed and stained for the myc-tag (E). Knockdown was confirmed by western blots in parallel samples in each experiment (F). Quantification of data was performed as described in Figure 1 (G). Scale bar represents 50 μm. n = 3; ^∗^p < 0.02. Error bars show SD.

We hypothesized that expression of Armus N terminus may result in enlarged vesicles by interfering with the normal function of endogenous Armus. However, expression of Armus_1–550_ did not interfere significantly with Rac1 activation (Figures 3A and 3B), and Armus coiled-coil domains (which interact with active Rac1) were not able to accumulate autophagosomes (Figure S3). These results excluded Rac1 titration as a mechanism for interfering with basal autophagy.

Surprisingly, in spite of the possible inhibition of lysosomal fusion (Figure 2), active Rab7 levels were not significantly affected by Armus_1–550_ expression (Figures 3C and 3D). A shorter fragment (Armus_1–169_) modestly activated Rab7 (Figures 3C and 3D), but this is unrelated to autophagy (Figure S3; data not shown). Further evidence suggested that Rab7 is not involved in the Armus_1–550_ phenotype. First, expression of the Armus RabGAP domain per se is not sufficient to accumulate vesicles (Figure S3). Second, depletion of endogenous Armus by distinct small interfering RNA (siRNA) oligos did not affect the number of enlarged vesicles in cells expressing Armus_1–550_ (Figures 3E–3G). We concluded that the function of endogenous Armus as a Rab7 GAP or as a Rac1 effector is not required for changes in basal autophagy triggered by Armus_1–550_.

### Armus Interacts Directly with LC3

**Figure 4:**
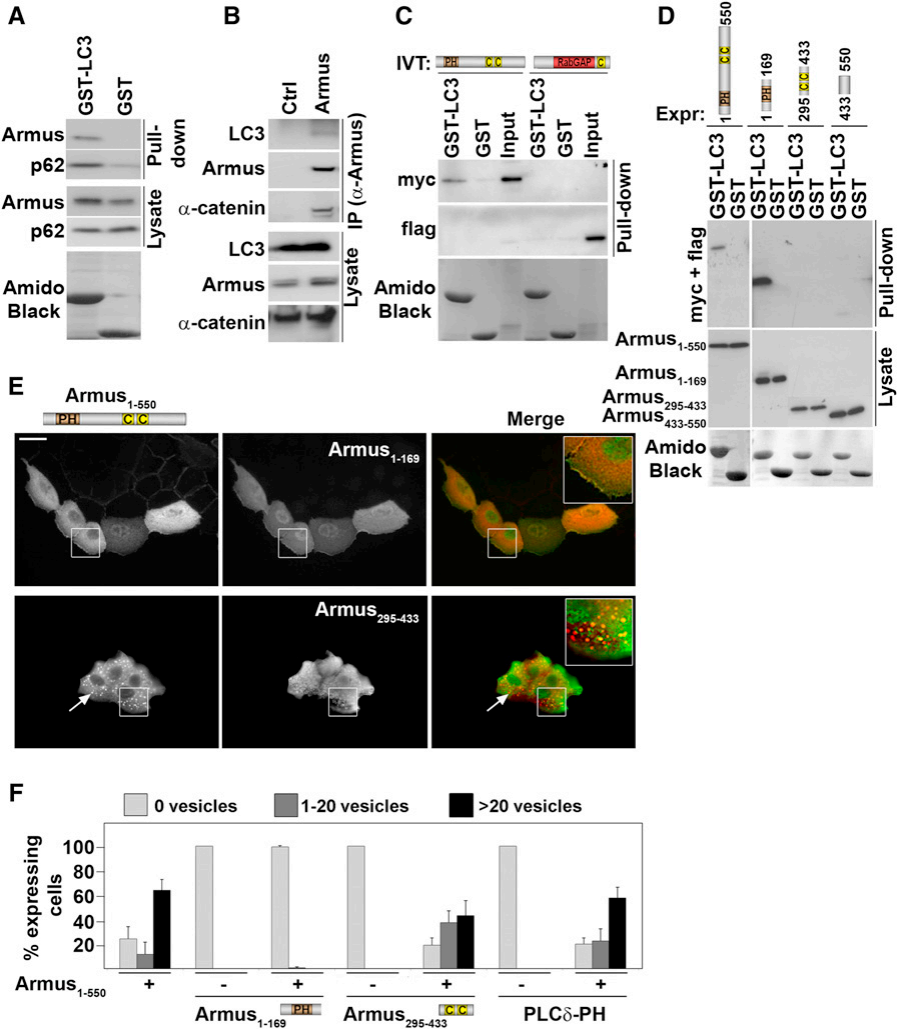
Armus Interacts with LC3 (A–D) Binding assays between Armus and LC3. Precipitated proteins and input were western blotted with antibodies against proteins shown on the left of each panel. Fusion proteins were stained with amido black. Top diagrams show Armus constructs used (transfection, Expr; or in vitro translation, IVT). (A) GST or GST-LC3 were used to pull down endogenous Armus from keratinocytes in full-nutrient conditions. (B) Endogenous Armus was immunoprecipitated (IP) from lysates in full-nutrient conditions. (C) IVT Armus N terminus (Armus_1–550_) or C terminus (Armus_547–928_) was incubated with GST or GST-LC3. (D) Different Armus mutants were transfected in keratinocytes and lysates incubated with GST or GST-LC3. (E and F) Armus_1–550_ was coexpressed with Armus_1–169_ or Armus_295–433_. Cells were fixed and stained for the respective tags. (F) Cells expressing different constructs (shown at the bottom of the graph) were quantified for the presence of autophagosomes as outlined in Figure 1B. Arrows show autophagosomes. Scale bar represents 50 μm. n = 3. Error bars represent SD. See also Figure S3.

An alternative explanation is that Armus could directly bind to and modulate the autophagy machinery. Glutathione S-transferase LC3 (GST-LC3) was able to pull down endogenous Armus from cell lysates (Figure 4A); conversely, endogenous Armus coimmunoprecipitated with endogenous LC3 (Figure 4B) in full-nutrient medium, implying that this interaction is significant for basal autophagy. GST-LC3 bound directly to the Armus N-terminal but not the C-terminal region (Figure 4C). LC3 interacted specifically with Armus_1–169_ and weakly to Armus_433–550_ (Figure 4D). Interestingly, Armus_1–169_ expression potently prevented enlarged autophagosomes induced by Armus N terminus expression (Figures 4E and 4F), but the control had no effect (PLCδ PH domain). It is feasible that Armus_1–169_ binding to LC3 prevents Armus_1–550_ recruitment to autophagosomes.

**Figure 5:**
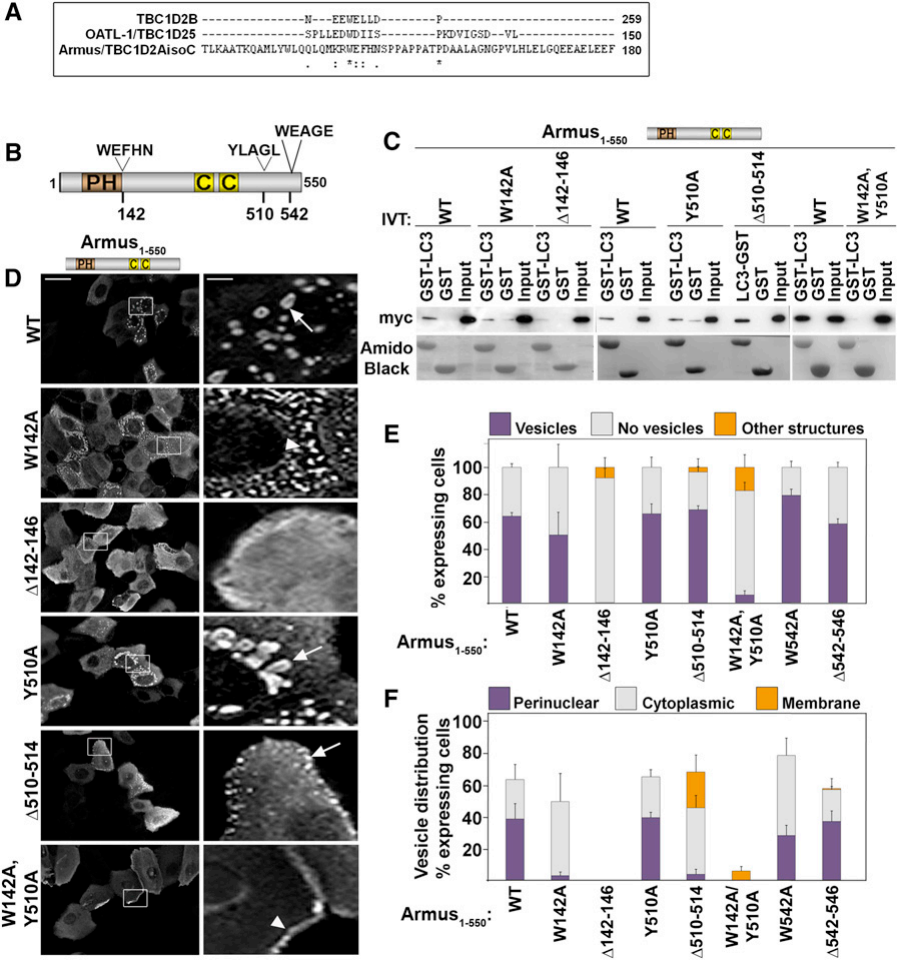
Armus Mutants Unable to Interact with LC3 Do Not Induce Autophagosome Accumulation in Full-Nutrient Medium (A) Alignment of Armus, TBC1D2B, and OATL1 sequences showing conservation of amino acids at the LIR). ^∗^ denotes show identity; · denotes conserved substitution; : denotes semiconserved substitution. (B) Armus_1–550_ diagram outlining different pentapeptides at positions 142, 510, and 542. Point mutations at the first amino acid and deletions of each pentapeptide were performed. (C) GST or GST-LC3 was incubated with in-vitro-translated (IVT) Armus_1–550_ mutants as shown in (B). Interacting proteins are revealed by probing for the myc-tag and fusion proteins are shown by amido black staining. (D) Different Armus_1–550_ mutants (shown on the left of images) were expressed in keratinocytes in full-nutrient medium, and cells were fixed and stained for the myc-tag. Zoom panels show the region highlighted by the white box. Arrows point to enlarged vesicles; arrowhead shows localization at cell-cell contacts. (E and F) Quantification of data shown in (D). (E) Percentage of expressing cells containing vesicles (purple), without vesicles (gray), or with other structures (orange). (F) Intracellular distribution of vesicles in keratinocytes expressing Armus mutants as perinuclear (purple), cytoplasmic (gray), or at the membrane (orange). Scale bar represents 50 μm or 7.7 μm for zoom. n = 3. Error bars show SD.

Thus, interaction with LC3 emerges as the likely mechanism for Armus interference with basal autophagy. Despite the low conservation of different LC3-interacting motifs (LIR) (Alemu et al., 2012; Behrends et al., 2010; Pankiv et al., 2010; Rozenknop et al., 2011), alignment of Armus, OATL (Itoh et al., 2011), and TBC1D2B (Behrends et al., 2010) identified sequence homology at amino acids 142–146 (WEFHN) (Figure 5A). Another pentapeptide could serve as potential LIR at 510–514 (YLAGL; Figure 5B), and WEAGE (amino acids 542–546) was used as control. The ability of Armus N terminus deletion or point mutants (single or in combination) to bind GST-LC3 (Figure 5C) or localize at autophagosomes (Figures 5D–5F) was evaluated. Residual LC3 interaction with single point mutants was observed, but binding to Armus^Δ142–146^ or Armus^W142A,Y510A^ (Figure 5C) was strongly reduced.

When expressed, Armus mutants showed striking defects on vesicle morphology and localization in full-nutrient medium (Figures 5D–5F). Armus^Δ510–514^ accumulated vesicles, although these were smaller and qualitatively different than those of wild-type Armus_1–550_. In contrast, Armus^W142A,Y510A^ and Armus^Δ142–146^ severely impaired vesicle accumulation (Figures 5D and 5E). In accordance to its residual LC3 binding, a single point mutation (Armus^W142A^ or Armus^Y510A^) was not sufficient to prevent accumulation (Figure 5C). Controls (Armus^W542A^ or Armus^Δ542–546^) showed a similar profile to Armus_1–550_ (Figures 5E and 5F; data not shown). Interestingly, rather than perinuclear vesicles as shown by wild-type, Armus^W142A^ and Armus^Δ510–514^ vesicles were dispersed in the cytoplasm and at the periphery, respectively (Figure 5F). We conclude that Armus N terminus has two LIR and that both sites cooperate for LC3 interaction. However, the region 142–146 appears to be the main LC3 binding site biochemically (Figures 4D and 5C) and functionally (Figures 5D and 5E). Mechanistically, Armus_1–550_ binding to LC3 seems sufficient to promote vesicle accumulation in basal autophagy.

### Armus Regulates Starvation-Induced Autophagy

**Figure 6:**
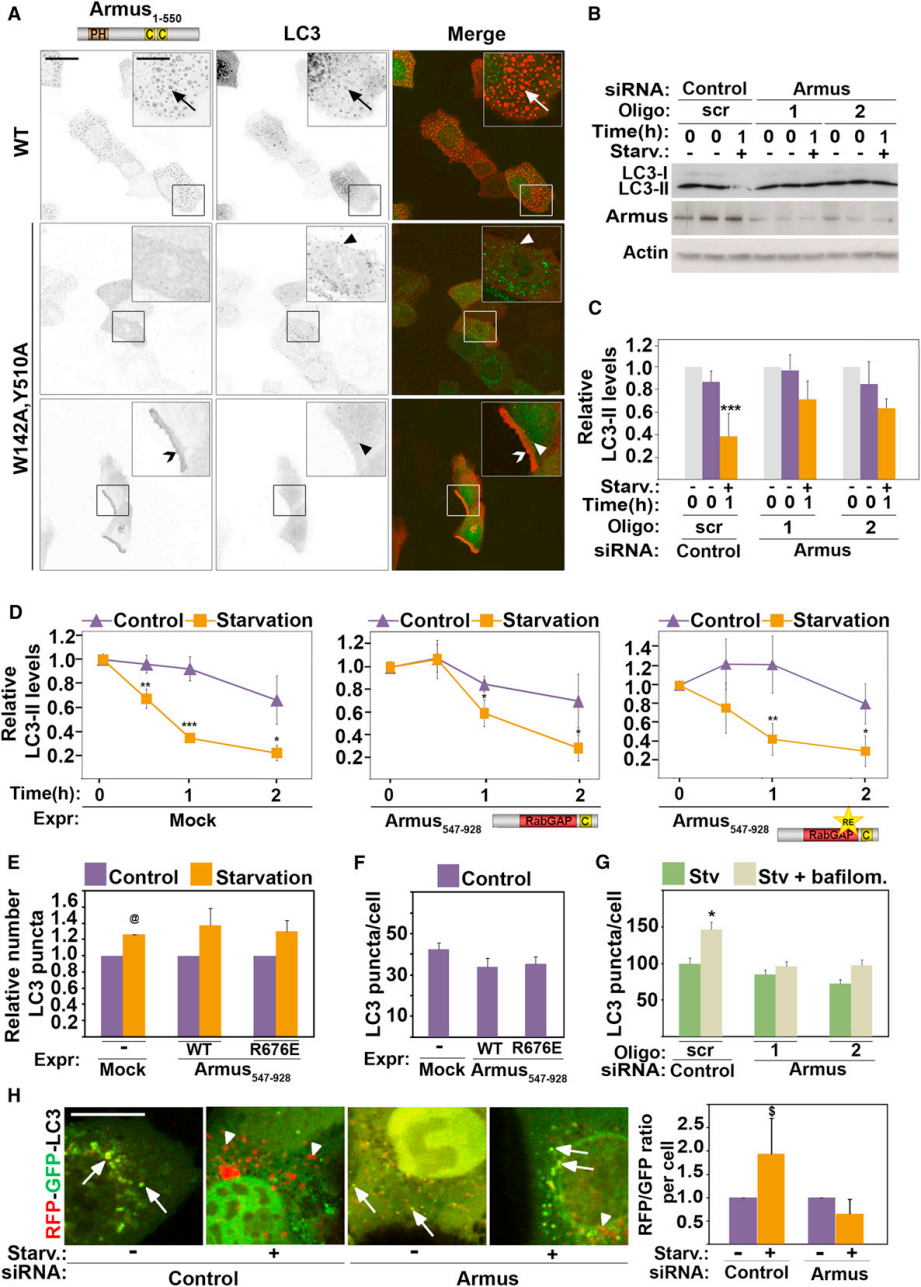
Armus Participates in Starvation-Induced Autophagy (A) Keratinocytes were transfected with GFP-LC3 and wild-type Armus or Armus^W142A,Y510A^, starved for 30 min, fixed, and stained for the tag. Inverted images are shown for clarity and merged images are shown on the right column. Inset shows amplification of the boxed area. Arrows show autophagosomes double labeled for Armus and LC3; arrowheads show LC3-puncta and open arrowhead points to Armus localization at the cell periphery. (B and C) Endogenous Armus is necessary for LC3 degradation. Keratinocytes were treated with control (scr) or two independent siRNA oligos against Armus. Cells were fed with full-nutrient medium 2 hr prior to the assay (T = 0) and maintained in the same medium for 1 hr (−, T = 1) or transferred to starvation medium for 1 hr (+, T = 1). (D–F) Starvation-induced LC3 degradation and number of LC3 puncta were monitored following transfection of different constructs: mock, Armus C-terminal region (Armus_547–928_) or catalytically inactive GAP (Armus_547–928_^R676E^). Cells were maintained in the same medium (control) or starved. (D) Endogenous LC3 protein levels were calculated and expressed relative to values at time zero for each group. (E) LC3 puncta were quantified and expressed relative to controls (nonstarved) in each group. (F) Basal levels of LC3 puncta are quantified in nonstarved cells (absolute number/cell). (G) Keratinocytes treated with Armus or scramble RNAi oligos were starved for 1 hr in the presence or absence of bafilomycin and the number of LC3 puncta quantified (see Experimental Procedures). (H) Tandem-fluorescent LC3 (Tf-LC3) was expressed in cells treated with Armus or control siRNA oligos and starved for 1 hr. Merged images (RFP and GFP) of starved and nonstarved cells are shown. Arrows show green/yellow autophagosomes, arrowheads point to red or acidic autophagosomes. The ratio of RFP and GFP dots per cell was calculated and expressed relative to control (scramble oligo nonstarved). Scale bar represents 16 μm or 6.4 μm for zoom (A) and 10 μm (H). n = 3; ^∗^p < 0.05; ^∗∗^p < 0.009; ^∗∗∗^p < 0.005; ^@^p < 0.00003. Error bars represent SD (C, D, G, and H) or SEM (E and F). See also Figure S4.

Our data suggest that binding to LC3 may be sufficient to recruit endogenous Armus to starvation-induced autophagosomes. Indeed, in contrast to wild-type, Armus^W142A,Y510A^ was not recruited to LC3 puncta triggered by starvation (Figure 6A). As a TBC/RabGAP regulating Rab7 cycling (Frasa et al., 2010), Armus could potentially facilitate fusion of autophagosomes with lysosomes during starvation, a necessary step for clearance of unwanted intracellular material. Our results strongly support this possibility. First, depletion of endogenous Armus reduced LC3 degradation following starvation (Figures 6B and 6C). Second, upon expression of wild-type GAP domain (Armus_547–928_), a delay in LC3 degradation was observed, but was not observed in controls (mock) or in Armus with impaired catalytic activity (R676E mutant; Figure 6D; Figures S4A–4C) (Frasa et al., 2010). Similar profile was observed for p62 degradation (Figures S4D–4F). Thus, depletion of Armus (which prevents Rab7 inactivation) or expression of Armus GAP domain (which forcibly inactivates Rab7) interferes with LC3 degradation, as Rab7·GTP-Rab7·GDP cycling is perturbed in both conditions.

Third, our data suggest that Armus does not regulate autophagosome nucleation, but rather regulates the later stages of autophagosome biogenesis: (1) basal and starvation levels of GFP-LC3 puncta were not inhibited by depletion of endogenous Armus or expression of Armus GAP domain (Figures 6F and 6G); (2) in starved cells without Armus protein, bafilomycin treatment did not further increase the number of LC3 puncta (Figure 6G), consistent with the fact that autolysosome formation was already inhibited; and (3) levels of acidification of LC3 puncta were compromised upon Armus depletion (Figure 6H).

**Figure 7:**
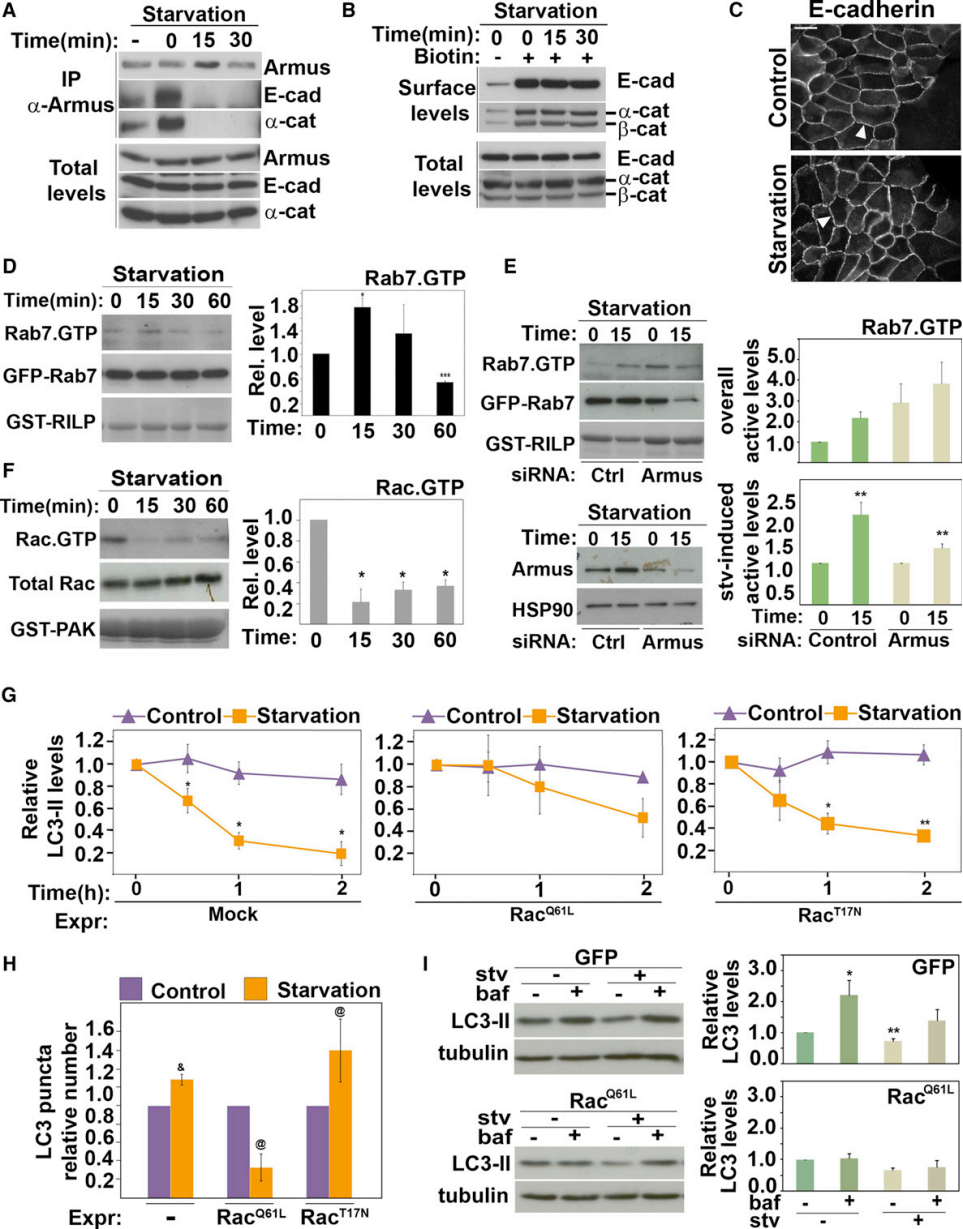
Rac1 Is Inhibited during Starvation-Induced Autophagy (A–C) Keratinocytes were starved for up to 30 min and (A) endogenous Armus was immunoprecipitated, or (B) cells were processed for biotinylation of surface proteins or (C) stained for E-cadherin. Lysates and precipitated samples were western blotted and probed for the proteins labeled on the right of each panel (A and B). (D–F) Keratinocytes (controls or Armus depleted) were starved for up to 60 min. Lysates were processed to detect active Rab7 (D and E) or active Rac1 (F). GST-fusion proteins were detected by amido black staining. Values were expressed relative to time zero (arbitrarily set as 1). (D and E) Lysates of cells expressing GFP-Rab7 were incubated with GST-RILP to pull down active Rab7 (Rab7·GTP). Levels of GFP-Rab7 (Total Rab7) and active Rab7 (Rab7·GTP) were detected with anti-GFP antibody. Active Rab7 values were corrected for total Rab7 and expressed relative to nonstarved samples (D), control scramble oligo in nonstarved cells (E, top graph), or nonstarved controls in each group (scramble or Armus RNAi; E, bottom graph). (F) Levels of active Rac1 (Rac·GTP) were determined using PAK-CRIB pull-down and probing with anti-Rac1. Levels of endogenous Rac1 (Total Rac) were quantified and used to calculate the relative amount of active Rac1. (G) LC3 degradation was monitored following transfection of activated Rac1 (Rac^Q61L^), dominant-negative Rac1 (Rac^T17N^), or mock. Cells were maintained in the same medium (control) or induced to starve for up to 2 hr (starved) by incubation in amino-acid-deficient medium. LC3 protein levels were calculated and expressed relative to LC3 at time zero in each group (n = 2). (H) Cells expressing GFP-LC3 and myc-Rac^Q61L^ or myc-Rac^T17N^ were starved for 15 min and then fixed and stained for the myc-tag. LC3 puncta was quantified (see Experimental Procedures) and expressed relative to the amount present in controls in each group (arbitrarily set as 1). (I) Cells expressing GFP or activated Rac were treated with bafilomycin during starvation for 1 hr and endogenous LC3 levels measured by blots. Values were corrected for tubulin levels and expressed relative to nontreated control in each group. n = 3; ^∗^p < 0.05; ^∗∗^p < 0.01; ^∗∗∗^p < 0.005; ^&^p < 0.0005; ^@^p < 0.02. Error bars show SD (D, F, G, and H) or SEM (E and I). See also Figure S4.

Lysosomal degradation of E-cadherin (Frasa et al., 2010) and LC3 are regulated by Armus, but it is unclear whether these two processes are interdependent. Starvation released endogenous Armus from cadherin complexes (Figure 7A), indicating that Armus is recruited to other intracellular compartments. E-cadherin surface levels or localization at junctions were not affected by starvation or lack of associated Armus (Figures 7B and 7C). We concluded that starvation does not perturb cell-cell adhesion within the time frame investigated, but rather recruits Armus away from cadherin complexes.

### Rab7 and Rac1 Activities Correlate Negatively during Starvation-Induced Autophagy

Given the known relationships between Rac1, Armus, and Rab7, we next sought to examine whether this function of Armus correlates with changes in Rab7 or Rac1 activity during autophagy. Rab7 was transiently activated by nutrient deprivation for 15 min (Figure 7D), indicating that Rab7 is cycling rapidly to allow autophagosome-lysosome fusion. Following Armus RNA interference (RNAi), overall levels of active Rab7 were higher than controls (scramble oligos, Figure 7E top graph), suggesting that Rab7 inactivation is compromised, a step required to release Rab7 from donor vesicles and lysosome fusion. Furthermore, the starvation-dependent increase in Rab7·GTP levels at 15 min was perturbed (Figure 7E, bottom graph), consistent with a defect in Rab7 cycling. Intriguingly, starvation induced a significant inactivation of Rac1 that persisted for up to 1 hr (Figure 7F). This is in contrast to cadherin degradation, as Armus is an effector of activated Rac (see below). We concluded that Rab7 regulation in autophagy requires endogenous Armus and the activation profiles of Rac1 and Rab7 inversely correlate during starvation.

Our data raise the possibility that Rac1 inactivation is necessary for autophagy to progress. Active Rac1 expression (Rac^Q61L^) delayed LC3 degradation upon starvation, while dominant-negative Rac1 (Rac^T17N^) had no effect (Figure 7G; Figures S4G–4I). However, in contrast to Armus RNAi (Figure 6G), upon Rac activation the number of LC3 puncta was significantly reduced during starvation for 15 min (Figure 7H). Upon treatment with bafilomycin, there was no further increase in LC3 levels when Rac1 was activated (Figure 7I). The results suggest that expression of active Rac1 potently interferes with autophagic flux, most likely at the step of LC3 puncta formation rather than at later stages.

**Figure 8:**
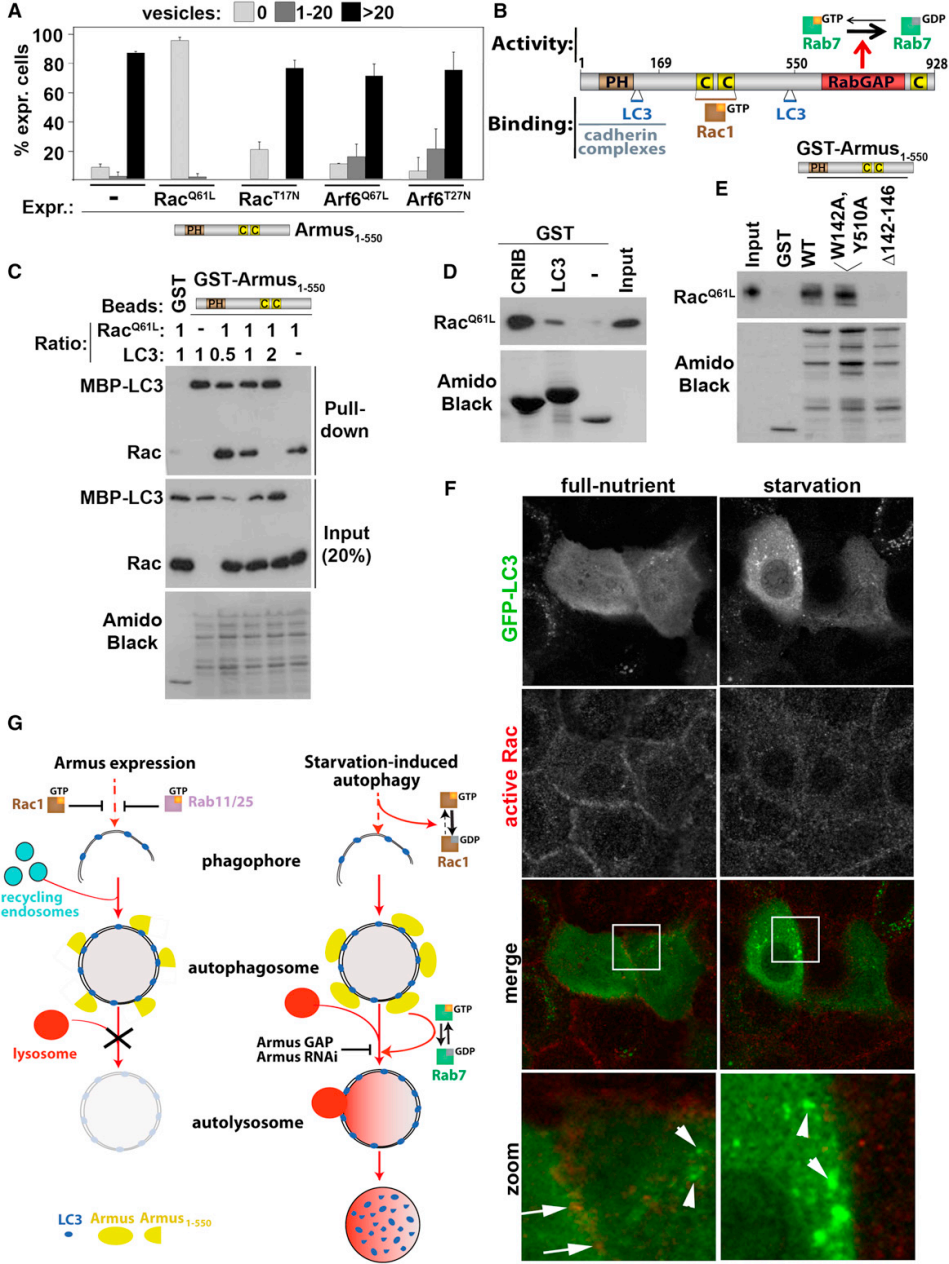
Molecular Interplay among Armus, Rab7, Active Rac1, and LC3 (A) Quantification of vesicles in keratinocytes microinjected with Armus_1–550_ by itself or in combination with active (Rac^Q61L^, Arf6^Q67L^) or dominant-negative (Rac^T17N^, Arf6^T27N^) small GTPases. The percentage of expressing cells showing no vesicles, 1 to 20 vesicles, or more than 20 vesicles was quantified for each condition. n = 3. Error bars show SEM. (B) Diagram showing full-length Armus, its binding partners and inactivation of Rab7·GTP into Rab7·GDP. Amino acids are noted on the top of the diagram. CC, coiled-coil domains; PH, Plekstrin homology domain; RabGAP, TBC/RabGAP domain. (C–E) Different binding assays were performed using purified proteins. Input and precipitated (pull-down) samples were probed antibodies against Rac1 or LC3. GST was used as negative control; amido black staining is shown. (C) GST-Armus_1–550_ was incubated with cleaved active Rac (Rac^Q61L^) with or without increasing amounts of MBP-LC3 in the molar ratio shown (top). (D) Cleaved active Rac1 (Rac^Q61L^) was allowed to interact with GST-LC3 or GST-PAK-CRIB as a positive control. (E) GST-Armus_1–550_ wild-type (WT) or mutants unable to interact with LC3 (W142A,Y510A and Δ142–146) were incubated with cleaved active Rac1 (Rac^Q61L^). (F) GFP-LC3 was expressed in cells and starvation was induced for 15 min. Following fixation in TCA, endogenous active Rac1 and GFP were stained with respective antibodies. Merged images and zoom are shown at the bottom. Arrows point to colocalization in full-nutrient medium, and arrowheads show LC3 puncta. (G) Summary of results. Armus expression leads to accumulation of enlarged autophagosomes via interaction with LC3 and displacement of endogenous Armus, leading to blockage of fusion with lysosomes. Increased fusion with other autophagosomes and/or recycling vesicles may also contribute to accumulation of enlarged autophagosomes. Activation of Rab11/Rab25 or Rac1 potently prevents autophagosome accumulation. Induction of autophagy by starvation inactivates Rac1 to allow endogenous Armus to be recruited to autophagosomes via a direct interaction with LC3. Armus localization at autophagosomes enables the regulation of Rab7 activity locally to mediate fusion with lysosomes and degradation. Upon starvation, depletion of endogenous Armus or expression of Armus GAP domain delays LC3 degradation, as both treatments interfere with Rab7 cycling. See also Figure S5.

Interestingly, coexpression of active Rac1 (Rac^Q61L^) with Armus_1–550_ in full-nutrient medium also prevented autophagosome accumulation (Figure 8A; Figures S5A and S5B), indicating that LC3 puncta formation and Armus recruitment did not occur in basal autophagy. Importantly, no interference with autophagosome accumulation was seen with Rac1 inhibition, activation, or inactivation of Arf6 (Figure 8A), a GTPase that regulates intracellular trafficking (D’Souza-Schorey and Chavrier, 2006). These data strongly indicate that (1) Rac1 activation potently inhibits basal and starvation-induced autophagy and (2) Rac1 acts upstream of Armus interaction with LC3, leading to reduced LC3 puncta.

A potential explanation is that Rac1 and LC3 may compete for binding with Armus as the Rac1 interaction site (Frasa et al., 2010) lies between the two identified LIR domains on Armus (Figure 8B). Indeed, increasing amounts of LC3 blocked the interaction of Rac1 to GST-Armus_1–550_ (Figure 8C). LC3 and active Rac can also interact directly (Figure 8D) and their binding sites on Armus did not overlap completely as Armus^W142A,Y510A^ interacted with active Rac1 in vitro (Figure 8E). Finally, endogenous active Rac1 (see methods) localized at cell-cell contacts in full-nutrient medium, as predicted. Active Rac1 also colocalized partially with GFP-LC3 in full-nutrient medium but was excluded from LC3 puncta during autophagy (Figure 8F). Total levels of active Rac were reduced upon nutrient depletion (Figure 8F), consistent with our previous biochemical data (Figure 7F).

Taken together, Rac1 activation interferes with autophagy via direct binding to LC3 and competing out other interaction partners, including Armus. Other Rac1-dependent signaling may also operate during autophagy. However, modulation of Rac1 activity during starvation did not interfere with phosphorylation levels of different molecules downstream of mTOR signaling cascade (Figures S5C–5G). Further work is required to identify potential pathways that can cooperate to inhibit autophagic flux following Rac1 activation.

## Discussion

The coordination of autophagy with trafficking and cytoskeletal remodeling is essential to allow autophagosome initiation, intracellular movement, and appropriate fusion with specific vesicles/organelles. Multiple Rabs and TBC/RabGAPs are predicted to regulate different steps in autophagosome biogenesis (Frasa et al., 2012). How their function is controlled in space and time has been the focus of intensive research (Behrends et al., 2010; Itoh et al., 2011; Longatti et al., 2012; Popovic et al., 2012). Here, we identify two regulators of autophagy, Rac1 and the TBC/RabGAP Armus, which are ideally placed to integrate different signaling events (Figure 8G). During starvation-induced autophagy, Armus regulates Rab7 cycling, autolysosome formation, and degradation of the autophagy protein LC3. In contrast, starvation strongly inhibits Rac1 and, conversely, Rac1 activation delays autophagic flux.

During basal autophagy, Armus expression accumulates autophagosomes that are orders of magnitude larger than starvation-induced autophagosomes. It is unlikely that changes in basal autophagy result from Armus aggregation, as interfering with a variety of signaling pathways prevents vesicle accumulation (Figure 8G). Strikingly, autophagosome accumulation is an autonomous property of Armus N terminus, as it does not require endogenous Armus. Instead, a direct binding to LC3 is the likely mechanism (Figure 8G). Armus N terminus expression may prevent recruitment of endogenous Armus to autophagosomes and lysosomal fusion, leading to abnormal size and number of basal autophagosomes. However, because LC3 has fusogenic properties (Nakatogawa et al., 2007; Weidberg et al., 2011), autophagosome homotypic fusion may be enhanced upon Armus expression and may also contribute to the phenotype observed.

Enlarged vesicles labeled with Armus contain recycling vesicles but are not acidic and do not recruit different lysosomal markers. Expression of Armus N terminus may promote the formation of a transient intermediate between recycling endosomes and autophagosomes. Although Armus does not inactivate Rab11a (Frasa et al., 2010), it is feasible that Rab11 could influence Armus function locally at autophagosomes. These data suggest that endogenous Armus may participate in Rab cascades leading to fusion with lysosomes. TBC/RabGAPs are ideally placed for such role as the same molecule can act as a Rab effector and inactivate distinct Rabs (Frasa et al., 2012). This interesting hypothesis warrants further investigation.

The above results inform us on the physiological role of endogenous Armus during nutrient withdrawal. It is unlikely that Armus regulates autophagosome nucleation, as Armus depletion does not prevent LC3 puncta formation or an increase in LC3 puncta during starvation. Instead, our data strongly support the idea that Armus regulates autolysosome biogenesis: (1) the direct interaction with LC3 localizes endogenous Armus at autophagosomes to facilitate Rab7·GTP hydrolysis, a necessary step to complete fusion with lysosomes (Figure 8); (2) during autophagy, increased Rab7·GTP levels are prevented by Armus depletion; (3) efficient degradation of LC3 requires endogenous Armus and its GAP activity; and (4) Armus is necessary for efficient acidification of autophagosomes. Thus, in a physiological setting, endogenous Armus modulates autophagic flux via its dynamic interaction with LC3, localized Rab7 regulation, and autolysosome formation.

The partial defects on autophagy induced by Armus depletion are consistent with redundancy in the regulation of Rab7 activity (Frasa et al., 2012). Rab7 localization and activity at late endosomes/autophagosomes are controlled by Rab7 effectors (Pankiv et al., 2010; Sun et al., 2010) and an exchange factor (Liang et al., 2008). Rab7 inactivation, however, is poorly characterized. Depletion of TBC1D15, a GAP for Rab7 (Peralta et al., 2010; Zhang et al., 2005), perturbs autophagic flux (Behrends et al., 2010), but the specific mechanism is unknown. TBC1D5, although predicted to inactivate Rab7, appears to regulate autophagosome formation (Popovic et al., 2012) and may potentially cooperate with TBC1D14 (Longatti et al., 2012). In contrast, depletion of OATL1 does not interfere with autophagic flux (Itoh et al., 2006, 2011), consistent with the fact that its substrate Rab33 (Itoh et al., 2008) regulates Golgi retrograde flow rather than lysosome function (Stenmark, 2009). Clearly, different TBC/RabGAPs and Armus have distinct functions during autophagy.

Armus modulates lysosomal fusion in two distinct cellular events: degradation of E-cadherin following EGF stimulation (Frasa et al., 2010) and LC3 during starvation (this work). However, Armus localization, binding partners, and upstream regulation are different in cell-cell adhesion and autophagy. First, at steady-state, a pool of Armus associates with cadherin complexes (Frasa et al., 2010) or with LC3. Upon nutrient deprivation, the integrity of cell-cell contacts is maintained, but Armus is relocalized to autophagosomes. Similar intracellular redistribution has been shown for other TBC/RabGAPs (Longatti et al., 2012; Popovic et al., 2012). Second, Rac1 activation is required for Armus-dependent E-cadherin degradation (Frasa et al., 2010), but in contrast, Rac1 is strongly inhibited by starvation. These results imply that a different regulator is responsible for activating Armus at autophagosomes.

We show here that Rac1 inhibition is essential for autophagic flux during starvation and potentially other stimuli (Zhu et al., 2011). Active Rac1 and LC3 compete for binding on neighboring domains in Armus. Such competition could prevent Armus localization to autophagosomes when Rac1 is activated and contribute to autophagy inhibition. Consistent with this finding, an active pool of endogenous Rac1 partially colocalizes with LC3 in full-nutrient medium, but not at LC3 puncta where Armus is recruited. However, this explanation is not the whole story, as Rac1 activation strongly reduces LC3 puncta formation and potently prevents accumulation of basal autophagosomes. These effects are distinct from Armus depletion and indicate that alternative Rac1 pathways upstream of Armus may be important.

Similar to Arf6 (Moreau et al., 2012), Rac1 signaling may also operate in early events during autophagosome biogenesis. Rac1 regulates a number of pathways that play a role in cell survival (Mack et al., 2011; Zoncu et al., 2011). Rac1 has been linked to the kinase mTOR (mammalian target of rapamycin) involved in cell-size regulation (Saci et al., 2011), tumor cell motility (Gulhati et al., 2011; Kim et al., 2011), or chemotactic migration (Hernández-Negrete et al., 2007; Kim et al., 2011). However, modulation of Rac1 activity does not regulate mTOR or its associated substrates during starvation. It is possible that other Rac1-dependent pathways may play a role in autophagy, and it will be important to explore these in future experiments.

In conclusion, distinct signaling downstream of different stimuli (cell scattering or starvation) regulate Armus localization (at junctions or autophagosomes) and lysosome-fusion events. We surmise that fine-tuning of Rac1 activity is required to allow Armus localization at autophagosomes and spatiotemporal coordination of Rab7 cycling to form autolysosome. Therefore, Rac1 and Rab7 functions are coordinated with efficient degradation of intracellular material during autophagy.

Our data have important implications for homeostasis and different pathologies, due to the essential cellular functions of Armus (Frasa et al., 2010), Rab7 (Mosesson et al., 2008; Stenmark, 2009), Rac1 (Vega and Ridley, 2008), and autophagy (Klionsky, 2007; Levine and Kroemer, 2008; Ravikumar et al., 2010b). It will be interesting to determine if Armus function is perturbed during the abnormal accumulation of autophagosomes seen in different diseases (Levine and Kroemer, 2008) or the autophagic response of tumor cells (Dikic et al., 2010; Eng and Abraham, 2011; Janku et al., 2011). How Armus participates in cancer has not yet been determined, yet Rab7 has an emerging role (Stenmark, 2009) and Rac1 has a well-established function in tumor proliferation and malignancy (Mack et al., 2011). Dissecting how signaling is orchestrated among these GTPases will provide exciting insights into autophagy regulation in health and disease.

## Experimental Procedures

### Cell Culture, Microinjection, and Transfection Procedures

Normal human keratinocytes isolated from neonatal foreskin (strain Sf, passages 3–6) were cultured as described previously (Braga et al., 1997). For experiments using starved cells, fresh medium was placed onto the cells for 2 hr prior to induction of autophagy before incubating with Earle’s balanced salt solution (EBSS) medium (Sigma) for up to 2 hr in different experiments. Autophagy was also induced by treatment with rapamycin (20 μM) for 1 hr in full-nutrient medium. To inhibit fusion of autophagosomes with lysosomes, transfected keratinocytes were incubated for 2 hr in full-nutrient medium in the presence of 50 μM vinblastine (Sigma) to disrupt microtubules. Alternatively, cells were starved in EBSS medium in the presence of 50 nM bafilomycin (Sigma) for 1 hr.

### Immunofluorescence and Microscopy

Immunofluorescence was carried out as previously described (Braga et al., 1997). For endogenous active Rac staining, a trichloroacetic acid (TCA) precipitation method was used that retains an insoluble pool of GTPases where activation takes place (Kamijo et al., 2006). TCA-insoluble, active Rac1 localized to lamellae (not shown) and at cell-cell contacts. Following fixation in 10% TCA for 15 min at room temperature, coverslips were washed three times in 30 mM glycine in PBS blocked in 3% BSA with 0.1% Triton X-100 in PBS for 1 hr and stained as normal.

Images were acquired with an Olympus Provis AX70 microscope, a SPOT RT monochrome camera, and SimplePCI software (Hamamatsu, Japan). Confocal images were acquired with a Leica DCS NT system or a Leica SP5 inverted system using Leica LCS Lite software. Images and figures were processed using Adobe Photoshop, Illustrator, or WCIF ImageJ software.

For electron microscopy, keratinocytes grown on gridded coverslips were fixed and processed for transmission electron microscopy as described elsewhere (Stinchcombe et al., 1995). The location of cells microinjected with Armus_1–550_ was recorded so they could be compared with cells in a noninjected area of the same coverslip. Glass coverslips were mounted cell side down on Epon stubs, and coverslips were removed by immersion in liquid nitrogen after polymerization overnight at 60°C. The grid was then readily visible on the surface of the Epon stub to allow location of the microinjected cells. Sections (70 nm) were stained with lead citrate and viewed in a Jeol 1010 transmission electron microscope.

### Protein Interactions

To detect in vivo interactions, keratinocytes were lysed (30 mM Tris [pH 7.5], 100 mM NaCl, 0.5% Triton, 5 mM EDTA, 1 mM dithiothreitol [DTT], 1 mM phenylmethanesulfonylfluoride, and 1 mM each protease inhibitors leupeptin, pepstatin, and pefabloc) and immediately frozen on dry ice. Lysates were defrosted quickly and centrifuged 5 min at 2,415 × *g* before incubation with different GST-tagged proteins on beads for 1 hr at 4°C. Alternatively, keratinocyte lysates were incubated with Protein-A beads (Sigma) for 1 hr to clear lysates followed by immunoprecipitation with anti-Armus antibody for 2 hr at 4°C. To investigate the regions important for LC3 binding, keratinocytes were transfected with different constructs and subjected to pull-down assay with GST-LC3 as described above.

To detect specific interactions, GST-tagged LC3 immobilized on beads was incubated with in-vitro-translated fragments of Armus or Armus mutants created by site-directed mutagenesis as described above in a total volume of 100 μl (10 mM Tris [pH 7.5], 350 mM NaCl, 1 mM DTT) for 30 min at 4°C. Beads were washed three times in 500 μl buffer (as above). To confirm interaction in vivo, different GST-tagged proteins on beads were incubated for 1 hr at 4°C with keratinocyte lysates (endogenous proteins or transfected with different constructs).

### In Vivo Activity

Determination of the levels of active Rab7 (Frasa et al., 2010) and active Rac1 (Betson et al., 2002) in vivo was carried out as described. Briefly, cells were starved for different amount of time (see cell culture) and lysates were prepared and incubated with GST-PAK-Crib or GST-RILP to pull down active forms of Rac1 and Rab7, respectively. Because of the low levels of endogenous Rab7, cells were transfected with wild-type GFP-Rab7 prior to the assays. Specific bands were detected with anti-Rac1 or anti-GFP antibodies. GST and GST-fusion protein loading were visualized by amido black staining (Sigma). For determination of active Rac and Rab7, levels of proteins associated with PAK-crib or RILP (GTP-bound or active pools) were expressed as a percentage of the total levels of proteins (endogenous Rac or GFP-Rab7). Values obtained for the control (no starvation) were arbitrarily set as 1.

## Supplemental Information

Document S1. Figures S1–S5 and Supplemental Experimental Procedures
