# Supplementary material for: The TBC/RabGAP Armus Coordinates Rac1 and Rab7 Functions during Autophagy
Source: Dev Cell. 2013 Apr 15;25(1):15–28. doi: 10.1016/j.devcel.2013.03.005 (PMC3898768; doi:10.1016/j.devcel.2013.03.005)
Supplement: Document S1. Figures S1–S5 and Supplemental Experimental Procedures [file mmc1.pdf]

Developmental Cell, Volume *25*

Supplemental Information

The TBC/RabGAP Armus Coordinates

Rac1 and Rab7 Functions during Autophagy

Bernadette Carroll, Noor Mohd-Naim, Filipe Maximiano, Marieke A. Frasa, Jessica McCormack, Mattea Finelli, Sigrid B. Thoresen, Louis Perdios, Reiko Daigaku, Richard E. Francis, Clare Futter, Ivan Dikic, and Vania M.M. Braga

Inventory of Supplementary Figures:

Fig. S1 shows controls for Fig.1: E-cadherin does not localize to Armus-labelled vesicles and enlarged vesicles are not a consequence of general protein aggregation.

Fig. S2 complements Fig. 2d. This figure further characterizes Armus-labelled vesicles with respect to endocytic markers and the phenotype obtained by activation the recycling Rabs Rab11 and Rab25 (quantification shown in Fig. 2f).

Fig. S3 maps the minimum regions of Armus able to promote enlarged vesicles (complement data shown in Fig.4e-f).

Fig. S4 shows Western blots used to quantify LC3 levels in graphs Fig. 6d and Fig. 7g and quantification of p62 levels in the presence of Armus GAP domain or catalytic dead mutant.

Fig. S5 shows signalling pathways that regulate the formation of enlarged vesicles labelled with Armus; quantified data is shown in Fig. 8a. It also addresses whether Rac1 function modulates mTOR signalling pathways.

Figure S1

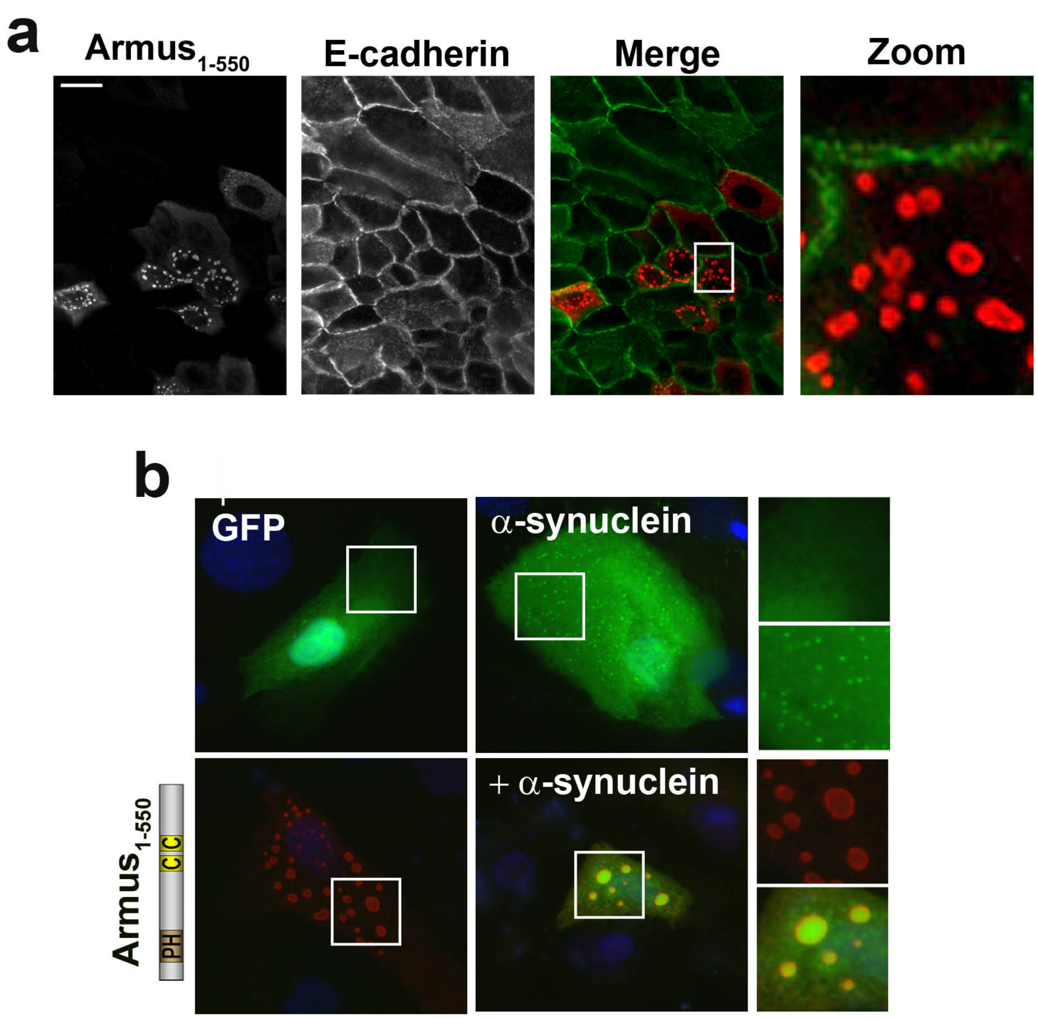
a
Armus1-550
E-cadherin
Merge
Zoom
b
GFP
α-synuclein
+ α-synuclein
Armus1-550
PH
CC

Figure S2

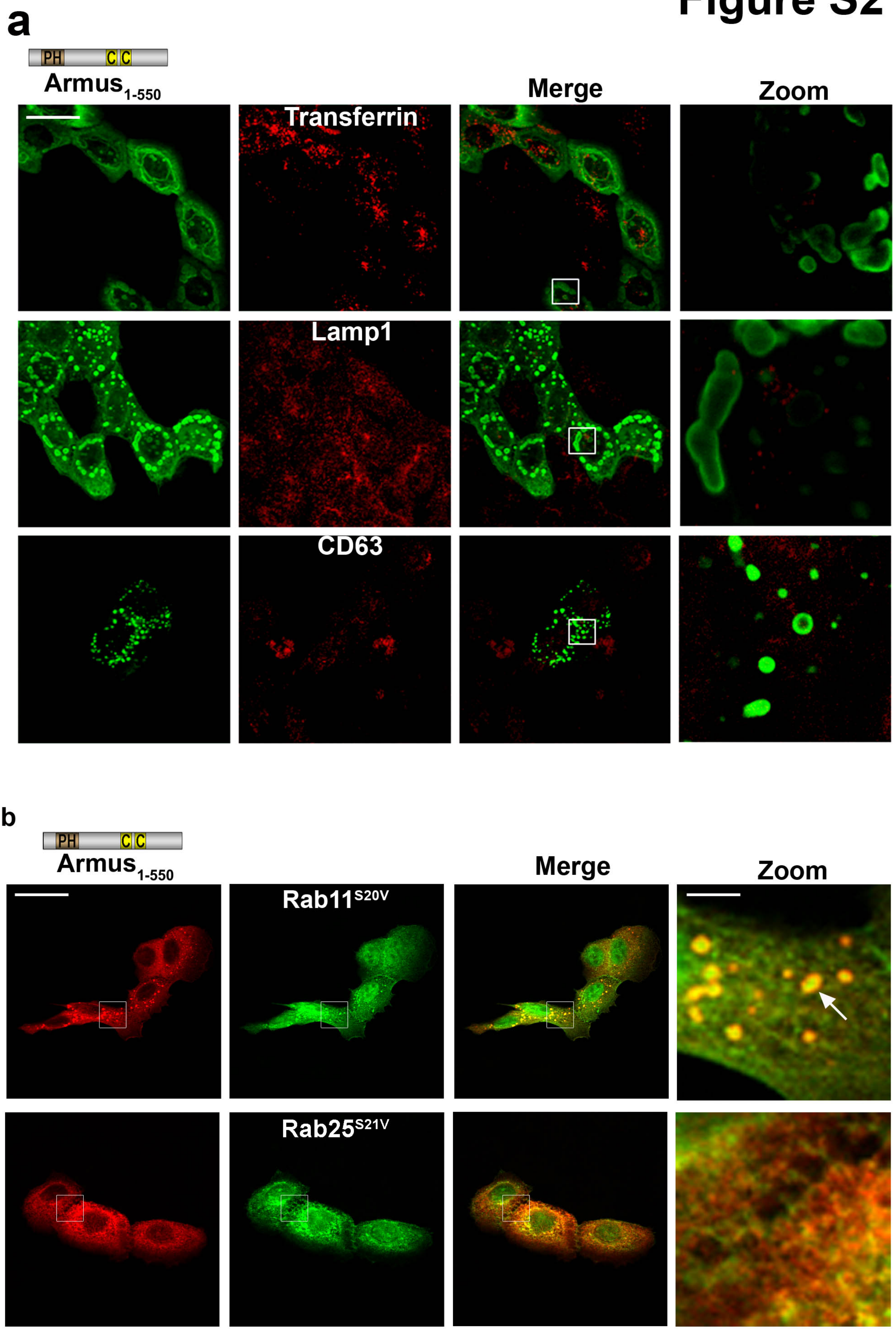
a
PH
C C
Armus1-550
Merge
Zoom
Transferrin
Lamp1
CD63
b
PH
C C
Armus1-550
Merge
Zoom
Rab11S20V
Rab25S21V

Figure S3

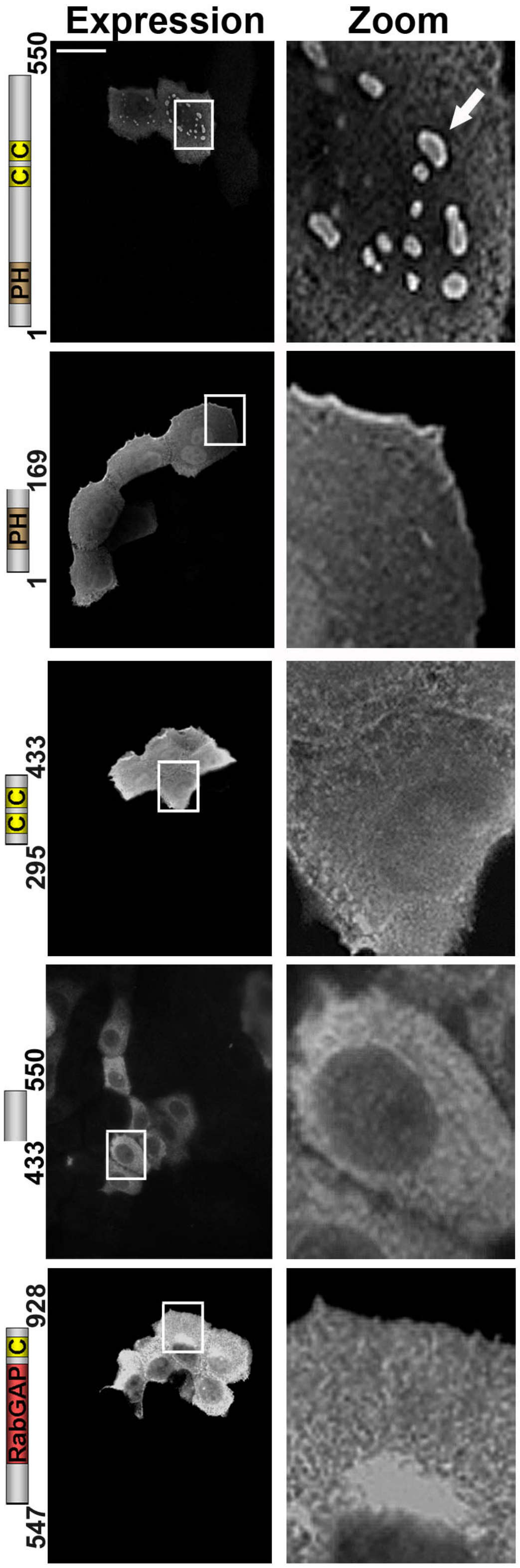

Expression
Zoom
550
C
C
PH
1
169
PH
1
433
C
C
295
550
433
928
C
RabGAP
547

Figure S4

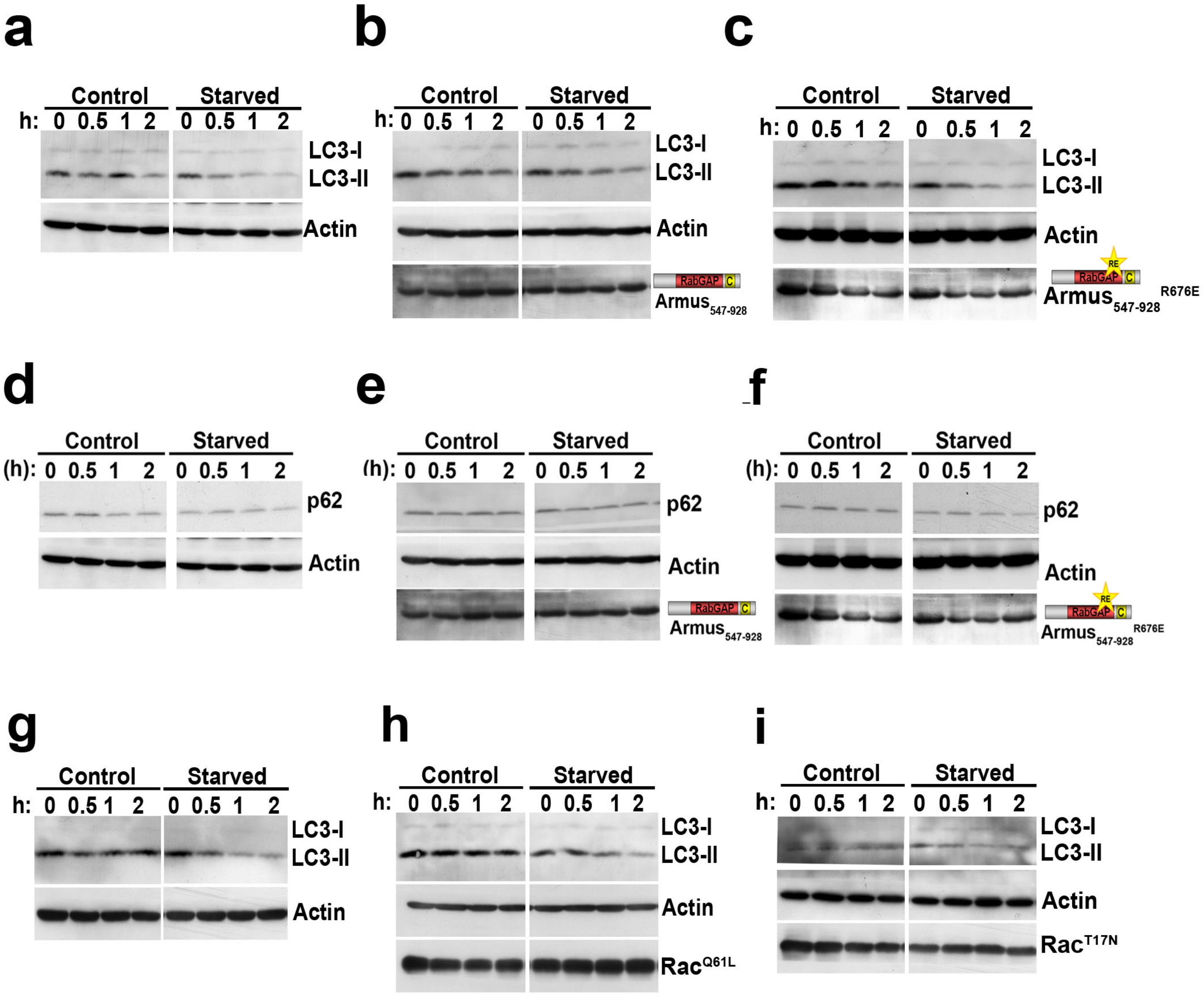

a
Control
Starved
h: 0 0.5 1 2 0 0.5 1 2
LC3-I
LC3-II
Actin
b
Control
Starved
h: 0 0.5 1 2 0 0.5 1 2
LC3-I
LC3-II
Actin
RabGAP C
Armus547-928
c
Control
Starved
h: 0 0.5 1 2 0 0.5 1 2
LC3-I
LC3-II
Actin
RE
RabGAP C
Armus547-928 R676E
d
Control
Starved
(h): 0 0.5 1 2 0 0.5 1 2
p62
Actin
e
Control
Starved
(h): 0 0.5 1 2 0 0.5 1 2
p62
Actin
RabGAP C
Armus547-928
f
Control
Starved
(h): 0 0.5 1 2 0 0.5 1 2
p62
Actin
RE
RabGAP C
Armus547-928 R676E
g
Control
Starved
h: 0 0.5 1 2 0 0.5 1 2
LC3-I
LC3-II
Actin
h
Control
Starved
h: 0 0.5 1 2 0 0.5 1 2
LC3-I
LC3-II
Actin
RacQ61L
i
Control
Starved
h: 0 0.5 1 2 0 0.5 1 2
LC3-I
LC3-II
Actin
RacT17N

Figure S5

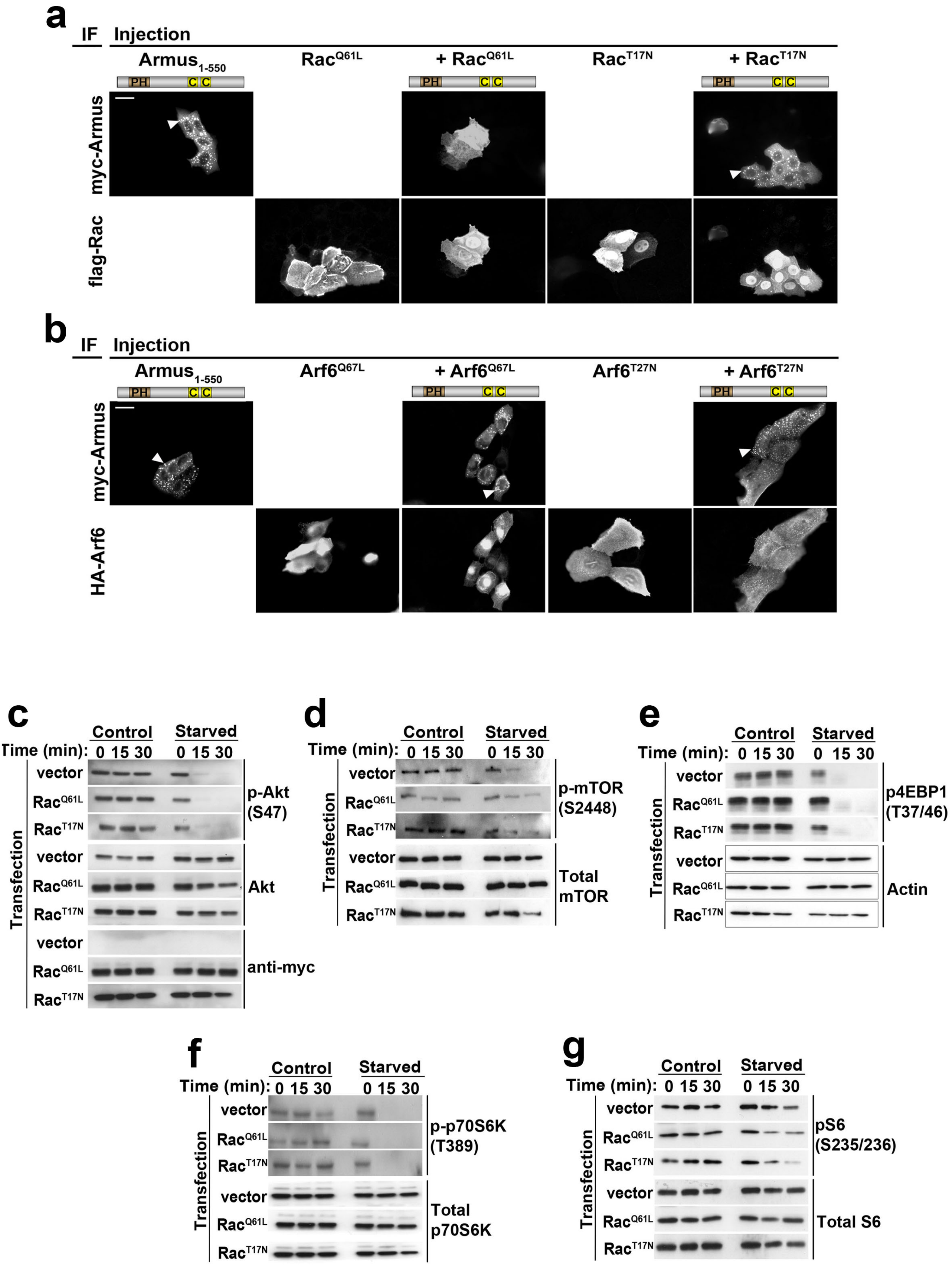

a
IF
Injection
Armus1-550
RacQ61L
+ RacQ61L
RacT17N
+ RacT17N
PH C C
myc-Armus
flag-Rac
b
IF
Injection
Armus1-550
Arf6Q67L
+ Arf6Q67L
Arf6T27N
+ Arf6T27N
myc-Armus
HA-Arf6
c
Control
Starved
Time (min): 0 15 30 0 15 30
Transfection
vector
RacQ61L
RacT17N
p-Akt (S47)
Akt
anti-myc
d
p-mTOR (S2448)
Total mTOR
e
p4EBP1 (T37/46)
Actin
f
p-p70S6K (T389)
Total p70S6K
g
pS6 (S235/236)
Total S6

Supplementary figures:

Figure S1, related to Figure 1: E-cadherin does not co-localise with Armus-induced vesicles. **a**, Keratinocytes were microinjected with $Armus_{1-550}$, and after expression for 3 hours, cells were fixed and stained for E-cadherin (green) and the myc-tag (red). **b**, α-synuclein (pEGFP-synuclein^{A53T}) was expressed alone or in combination with $Armus_{1-550}$ in full-nutrient medium. Cells were stained for the tags and GFP expression was used as control. Right panels show enlargement of area highlighted by white rectangles. n=3. Scale bar=50μm or 7.7μm for zoom.

Figure S2, related to Figure 2: a, Keratinocytes were injected with $Armus_{1-550}$ and after 3 hours, cells were fixed and stained for the tag and antibodies against Lamp1 or CD63. Alternatively, keratinocytes were allowed to uptake Texas-Red-labelled transferin for 30 minutes. **b**, Keratinocytes were microinjected with myc-$Armus_{1-550}$ alone or in combination with GFP-tagged versions of constitutively active Rab11 (Rab11^{S20V}) or Rab25 (Rab25^{S21V}). Quantification of the vesicles is shown in Fig.2f. Arrow points to autophagosome containing Armus and active Rab11. Last column on the right shows amplification of the region marked by the white rectangle of merged images. n=3. Scale bar=16μM and 4μM for zoom.

Figure S3, related to Figure 4: Enlarged vesicles are induced by Armus N-terminal region, but not truncation mutants. Different Armus truncation mutants as shown by the diagrams on the left of panels were injected in keratinocytes. Cells were fixed and stained for the tag. Right panels show enlargement of area highlighted by the white boxes (zoom). Arrow points to an enlarged vesicle labelled with Armus. n=3. Scale bar=50μm or 7.7μm for zoom.

Figure S4, related to Figures 6 and 7: Starvation-induced LC3 degradation is assessed following transfection of different Armus constructs. Cells were maintained in the same medium (control) or induced to starve for up to 2 hours (starved) by transferring to amino acid-deficient medium. Lysates were prepared and probed for endogenous LC3 (**a**-**c**, **g**-**i**) or p62 (**d**-**f**), expression tags and actin as shown on the right of each panel. The following constructs were expressed: **a**,**d**,**g**, mock; **b**,**e**, Armus C-terminal region ($Armus_{547-928}$); **c**,**f**, catalytically inactive GAP ($Armus_{547-928}^{R676E}$); **h**, activated Rac (Rac^{Q61L}) or **i**, dominant negative Rac (Rac^{T17N}). Quantification of LC3 levels is shown in **Fig**.6d and **Fig**.7g, respectively. n=2 (g-i) or n=3 (a-f)

Figure S5, related to Figure 8: Rac activation, but not Arf6, prevents accumulation of enlarged vesicles by Armus expression. a - b, Keratinocytes were microinjected with $Armus_{1-550}$ by itself or in combination with: constitutively active (Rac^{Q61L}, $Arf6^{Q67L}$) or dominant-negative (Rac^{T17N}, $Arf6^{T27N}$) small GTPases. Cells were fixed and stained for the respective tags. Quantification of the percentage of cells containing different number of vesicles is shown in **Fig**.8a. Arrowheads show Armus-labelled enlarged vesicles. **c** - **g**, Rac signalling does not perturb the levels or phosphorylation of autophagy upstream regulators. Keratinocytes were transfected with empty vector, activated (myc-Rac^{Q61L}) or dominant-negative Rac (myc-Rac^{T17N}) and maintained in the same medium or starved for up to 30 minutes. Lysates were prepared and probed for the myc-tag and the following proteins and their phosphorylated counterparts: **c**, Akt; **d**, mTOR, **e**, phospho4EBP1; **f**, p70SK and **g**, S6. Scale bar=50μM. n=3.

Supplementary material - Methods

DNA constructs

The following constructs were used: pRK5-myc-Rac^{Q61L} and pGEX-2T-Rac^{Q61L} (constitutively active; A.Hall, Memorial Sloan-Kettering Cancer Center, New York); pCS2-myc-Rac^{T17N} (dominant-negative); pCS2-HA-Arf6^{Q67L} (constitutively active) and pCS2-HA-Arf6^{T17N} (dominant-negative).(Frasa et al., 2010) Constitutively active Rab11 (pEGFP-C2-Rab11a^{S20V}) and Rab25 (EGFP-C2-Rab25^{S21V}; both from J. Goldenring (Vanderbilt University, Nashville, USA); pEGFP-Rab7 (wild-type), pEGFP-Rab5^{Q79L} (constitutively active) (both from M. Seabra; Imperial College London, UK) and pEGFP-PLC$\delta 1$–PH (aa 2-170) were used. Other gifts include pEGFP-C3-LC3 and Tf-LC3 (mRFP-EGFP-LC3) (T.Yoshimori, Osaka University, Japan), pGEX2T-PAK-CRIB and RILP in pGEX-6P vectors (C. Bucci, Università del Salento, Italy) and pEGFP-α-synuclein^{A53T} (D. Rubinsztein, Cambridge Institute for Medical Research, UK). The ATG8 family member LC3A was also used in pGEX-4T1 and pMAL-C2X vector.

Armus (accession number Q9BYX2-1) constructs previously described (Frasa et al., 2010) include full-length Armus$_{1-928}$ (Venus- and RFP-tagged); N-terminus Armus$_{1-550}$ (in pRK5myc and pGEX-6P1); PH domain (pRK5flag-Armus$_{1-169}$); C-terminus Armus$_{547-928}$ (pRK5flag- and pGEX-4T3); catalytically impaired Armus GAP domain (pRK5flag-Armus$_{547-928}^{R676E}$). Smaller fragments were subcloned into flag- or myc-tagged pRK5 (Armus$_{295-433}$, Armus$_{369-433}$ and Armus$_{433-550}$). To generate Armus mutants unable to bind LC3, site-directed mutagenesis was performed in pRK5myc-Armus$_{1-550}$ to generate deletions (Armus$^{\Delta 141-145}$, Armus$^{\Delta 509-513}$, Armus$^{\Delta 542-547}$) or point mutations (Armus^{W141A}, Armus^{Y509A}, Armus^{W542A} and double mutations Armus^{W141A,Y509A}). Primer sequences used in mutagenesis are available upon request.

Transfections and RNAi:

Microinjection experiments were carried out as detailed previously (Braga et al., 1997; Lozano et al., 2008). cDNA transfections were done with TransIT-Keratinocyte transfection reagent (MirusBio) or JetPRIME (Polyplus transfection) as per company instructions. During co-expression of different plasmids, the amount of each plasmid was optimized to obtain equivalent levels of expressed proteins. For cDNA microinjections in siRNA-treated cells, injections were carried about 70 hours of post-siRNA transfection and cells incubated for further 3 hours. For siRNA transfections, keratinocytes were transfected with oligonucleotides targeting Armus (Frasa et al., 2010) or a control, scramble oligonucleotide (Dharmacon) using RNAifect (Qiagen) (Lozano et al., 2008) or Interferin (Polyplus transfection) as described. Where cDNA transfections were performed in siRNA-treated cells, the cDNA transfection protocol was carried out 48 hours or 4 hours post-siRNA transfection and incubated for further 24 hours for cells in low calcium or standard medium respectively. Similar results were obtained in either condition.

Antibodies and conjugates:

Rabbit polyclonal antibodies used are anti-Armus (Frasa et al., 2010), anti-Rab11a and anti-Rab25 (from J. Goldenring) and anti-CD63 (IB5; gift from M. Marsh, University College London, UK) or purchased from Cell Signalling: anti-Rab7, anti-phospho mTOR (Ser2448), anti-mTOR (7C10), anti-phospho S6K (Ser 235/236, 91B2), anti-S6K (5G10), anti-phospho p70S6K (Thr389), anti-p70S6K (49D7), anti-phospho Akt (Ser47 3), anti-Akt (11E7), anti-phospho 4E-BP1 (Thr37/46, 236B4). Rabbit anti-GFP was purchased from Abnova.

Mouse monoclonal antibodies used were anti-E-cadherin (HECD-1, CR-UK), anti-myc (9E10, Upstate), anti-flag (M2, Sigma), anti-Rac (23A8, Upstate), anti-actin (C4, MP Biomedicals), anti-β-tubulin (Tub 2.1, Sigma), anti-LC3 (5F10, Nanotools), anti-p62 (clone 3, BD Biosciences), anti-GFP (3E1, CRUK) and anti-Lamp1 (clone 25, BD Pharmingen or Abcam). Monoclonal rat anti-E-cadherin (ECCD2) was purchased from Zymed Laboratories and anti-HA epitope (3F10) from Roche Applied Science.

Transferrin A568 (Molecular Probes) was added to the medium for 30 minutes prior to cell fixing. To label lysosomes, cells were incubated with 50nM lysotracker red (Invitrogen)

for 30 minutes followed by 2 hours chase. Secondary antibodies were bought from Jackson Immuno Research Laboratories (fluorescent-conjugates, Stratech Scientific) or Pierce (HRP-conjugates).

Production of fusion proteins

GST fusion proteins for $Armus_{1-550,}$ RILP and PAK-CRIB were produced as previously described (Betson et al., 2002; Frasa et al., 2010) and purified using standard techniques. Beads were washed 4 times in 50mM Tris pH7.5, 100mM NaCl, 5mM DTT, 1mM PMSF buffer re-suspended in 60% glycerol in the same buffer and kept as small aliquots at -80°C. Different constructs were *in vitro* translated using a rabbit reticulocyte coupled kit as per manufacturer's instructions (SP6 promoter, Promega).

Western blots

For detection of phosphorylated proteins involved in mTOR signalling pathway, keratinocytes were lysed (30mM Tris pH7.5, 100mM NaCl, 0.5% Triton, 5mM EDTA, 1mM DTT, 1mM each protease inhibitors (leupeptin, pepstatin, pefablock and PMSF), 50mM NaF, 1mM Na-orthovanadate, 20mM β-glycerophosphate, 30mM Na-pyrophosphate) and centrifuged for 5 minutes at 13,148Xg. Sample buffer was added to supernatant and samples were incubated at 100°C for 5 minutes followed by electrophoresis and Western blotting. For detection of LC3, keratinocytes were lysed (30mM Tris pH7.5, 100mM NaCl, 0.5% Triton, 5mM EDTA, DTT and protease inhibitor cocktail as above) and frozen immediately on dry ice. Lysates were defrosted quickly and centrifuged 5 minutes at 2415X*g*. Samples were incubated at 65°C for 5 minutes followed by electrophoresis in SDS-PAGE, transferred to membranes and signal detected with ECL plus Western blotting detection kit (GE Healthcare).

Quantifications:

Protein levels were detected by scanning multiple exposures of X-Ray films obtained by Western blots to ensure linear range of the response. Specific bands were quantified using WCIF ImageJ software. Quantification of LC3 levels from Western blots was performed following expression of different constructs or Armus RNAi and normalised for actin levels in each sample (internal control). LC3 levels at time zero was arbitrarily set at 1 for each group (control or starved) and subsequent time points expressed relative to time zero in each group. Statistical analysis compared the changes in LC3 levels at different time points when compared to time zero in controls or starved samples. Following expression of different Armus constructs, LC3 levels were expressed relative to empty vector (arbitrarily set as 1).

Quantification of the number of vesicles accumulated by $Armus_{1\text{-}550}$ was carried out by scoring cells expressing different constructs into three groups: cells containing no vesicles, 1-20 and more than 20 vesicles. Two quantitative analyses were performed to characterize Armus mutants unable to interact with LC3. First, expressing cells were quantified according to the appearance of vesicles (no vesicles, vesicles or other structures – tubular, elongated, etc). Second, the localization of vesicles in expressing cells was scored as cytoplasmic, perinuclear or close to the plasma membrane.

Quantification of LC3 puncta in Rac1 or GFP-LC3 expressing cells was carried out using WCIF ImageJ software. Briefly, a mask was made of the transfected cells and was used to subtract the background from the GFP-LC3 channel to ensure only LC3 puncta in expressing cells were included in the analysis. LC3 puncta (within an optimised size range) in the segmented image were counted using the 'particle counting' function and expressed as number of particles per expressing cell. Analysis of Tf-LC3 puncta was performed by quantification of the number of mRFP and EGFP puncta per cell, calculating the ratio mRFP/EGFP and normalising for the controls (arbitrarily set as 1). Statistical analysis was performed by Anova or student's T-test and p values shown in figure legends.

References

Betson, M., Lozano, E., Zhang, J., and Braga, V.M.M. (2002). Rac activation upon cell-cell contact formation is dependent on signaling from the epidermal growth factor receptor. J Biol Chem *277*, 36962-36969.

Braga, V.M.M., Machesky, L.M., Hall, A., and Hotchin, N.A. (1997). The small GTPases Rho and Rac are required for the establishment of cadherin-dependent cell-cell contacts. J Cell Biol *137*, 1421-1431.

Frasa, M.A., Maximiano, F., Smolarczyk, K., Francis, R.E., Betson, M.E., Lozano, E., Goldenring, J.R., Seabra, M.C., Rak, A., Ahmadian, M.R., *et al.* (2010). Armus is a Rac1 effector that inactivates Rab7 and regulates E-cadherin degradation. Curr Biol *20*, 198-208.

Lozano, E., Frasa, M.A., Smolarczyk, K., Knaus, U.G., and Braga, V.M. (2008). PAK is required for the disruption of E-cadherin adhesion by the small GTPase Rac. J Cell Sci *121*, 933-938.
